# Supplementary material for: Single-cell analysis of peripheral blood from high-altitude pulmonary hypertension patients identifies a distinct monocyte phenotype
Source: Nat Commun. 2023 Mar 31;14:1820. doi: 10.1038/s41467-023-37527-4 (PMC10066231; doi:10.1038/s41467-023-37527-4)
Supplement: Supplementary file 1 — Supplementary Information [file 41467_2023_37527_MOESM1_ESM.pdf]

Inventory of Supplementary tables and figures

Supplementary Table 1. Characteristics and PBMC fraction in the validation cohort.
Supplementary Table 2. The differentially expressed gene list of three subcluster of monocytes.
Supplementary Table 3. The correlation coefficient of clinical parameters and fractions of various PBMC cell types.
Supplementary Table 4. The correlation and P-value of clinical parameters and expression of HIF1-α and its target gene VEGFA.
Supplementary Table 5. The clinical parameters of PH samples.
Supplementary Table 6. Primers for real-time PCR.
Supplementary Table 7. siRNA sequences for *CD28*, *SPON2* and *TBX21*.

Supplementary Figure 1. The immune landscape of PBMC samples from patients with HAPH.
Supplementary Figure 2. The immune landscape of PBMC samples from patients with HAPH.
Supplementary Figure 3. Differential immune cell composition in the periphery of patients with HAPH.
Supplementary Figure 4. Differentially expressed genes (DEGs) in cluster 0 (C0) monocyte between HAPH patients and controls.
Supplementary Figure 5. Differentially expressed genes (DEGs) in cluster 1 (C1) monocyte between HAPH patients and controls.
Supplementary Figure 6. Differentially expressed genes (DEGs) in cluster 2 (C2) monocyte between HAPH patients and controls.
Supplementary Figure 7. Gene Ontology analysis (biological process) of the down-regulated genes in each monocyte subsets.
Supplementary Figure 8. Gene Ontology analysis (biological process) of the up-regulated and down-regulated genes in each cell type in comparison of HAPH with control group.
Supplementary Figure 9. Cell–cell communication networks between C1 monocyte and other peripheral blood cell types using CellPhoneDB.
Supplementary Figure 10. Cell–cell communication networks between C2 monocyte and other peripheral blood cell types using CellPhoneDB.
Supplementary Figure 11. Validation of gene expression in lung tissues of the mice model.
Supplementary Figure 12. Validation of protein expression in lung tissues of the mice model.
Supplementary Figure 13. Validation of protein expression in RAW264.7 monocytes/macrophages and THP-1 monocytes.
Supplementary Figure 14. Function assessments of TBX21 and SPON2 using a co-culture model.
Supplementary Figure 15. Identification of gene knockdown efficiency.
Supplementary Figure 16. Hif-1α (HIF-1α) expression in lung tissues of the PH mice and its expression in response to hypoxia-mimetic agent.

Supplementary Figure 17. Intact uncropped immunoblotting bands for Hif-1α (HIF-1α) expression in lung tissues of the PH mice and cells in response to hypoxia-mimetic agent.

Supplementary Figure 18. Differential immune cell composition in the circulation of patients with HAPH or PH.

Supplementary Figure 19. tSNE plots of individual samples in HAPH (H) and Control (C) group (a) and in HAPH (H) and PH (P) group (b).

Supplementary Figure 20. Monocyte clusters in PBMCs from patients with HAPH, PH and controls.

Supplementary Table 1. Characteristics and PBMC fraction in the validation cohort

ID	Group	Gender	Age	Altitude (m)	CD4/0.0% CD3+CD4+	CD8/0.0% CD3+CD8+	NK/0.0% CD3- CD16+CD56+	C0	C1	C2
C6	Controls	Female	47	3194.97	30.8	25.1	17.7	49.3	23	7.62
C7	Controls	Female	55	3194.97	32.6	16.2	13.1	45.7	30.9	8.61
C8	Controls	Male	48	3194.97	40.3	15.5	5.73	68.1	32.9	9.42
C9	Controls	Male	65	3194.97	40.6	11.4	8.17	61.9	26	5.09
C10	Controls	Female	50	3194.97	33.1	18.1	15.3	64.9	37.9	7.21
C11	Controls	Female	32	3109.72	13.9	30.7	16.6	55.5	17.2	11.8
C12	Controls	Female	64	3194.97	32.6	22.7	6.52	81.5	12	1.7
C13	Controls	Female	63	3109.72	38.3	16.1	16.5	68.3	33.1	9.39
C14	Controls	Male	55	3194.97	34.6	21.8	33.9	50	5.7	4.54
C15	Controls	Female	44	3194.97	36.7	19.8	15	68.9	12.1	3.35
H1	Controls	Female	67	3194.97	29.7	25.7	7.86	73.9	30.9	6.92
H2	HAPH	Female	76	3194.97	39.6	18.9	26.8	57.8	35	7.88
H3	HAPH	Female	51	3194.97	38.2	20.5	34.1	50	47.2	19
H4	HAPH	Female	60	3194.97	37.5	20	14.5	63.6	30.9	10.3
H5	HAPH	Male	77	3194.97	27.4	25.4	27.8	59.6	38.4	9.21
H6	HAPH	Male	72	3109.72	53	8.54	35.5	62.5	51	7.51
H7	HAPH	Female	57	3194.97	38.7	16.3	23.5	50.1	27.7	17.9
H8	HAPH	Female	48	3212.04	34.9	20	31.3	61.9	27.8	13.5
H9	HAPH	Female	44	3212.04	39.7	15.1	18.7	38.6	47.4	13
H10	HAPH	Female	56	3194.97	41.1	17.5	3.18	70.4	48.1	9.25
H11	HAPH	Female	59	3194.97	38.6	19.3	9.15	48.7	18.5	11.7

Supplementary Table 2. The differentially expressed gene list of three subcluster of monocytes

Moncyte_C0	Moncyte_C1	Moncyte_C2
S100A9	PPBP	IL32
CD14	CD68	NKG7
S100A8	FCGR3A	GNLY
LYZ	MS4A7	CTSW
S100A12	LST1	CST7
VCAN	MYL9	CD3E
MS4A6A	IFITM3	IFITM1
CSTA	RHOC	GZMH
MNDA	TIMP1	GZMB
FCN1	TPM1	PRF1
NFKBIA	PECAM1	GZMA
NCF1	TREML1	CD69
FOS	SMIM25	CCL5
TYMP	TPM4	FGFBP2
LGALS2	HIST1H2AC	PCED1B-AS1
CEBPD	FAM110A	CD7
BLVRB	ITGA2B	TRBC2
PLBD1	SERPINA1	CD247
MAFB	WARS	GZMM
VIM	C19orf33	KLRB1

Supplementary Table 3. The correlation coefficient of clinical parameters and fractions of various PBMC cell types

	B-cells	CD4+_ T-cells	CD8+_ T-cells	Memory_ B-cells	Monocytes	naive_ B-cells	NK_cells	Tregs	C0	C1	C2
Age	0.043	-0.235	-0.658 *	-0.245	0.284	-0.182	0.417	0.259	-0.126	0.011	0.081
Systolic blood pressure (SBP)	-0.649*	0.427	0.235	0.287	-0.494	-0.347	0.203	-0.144	0.088	0.322	-0.147
Diastolic blood pressure (DBP)	-0.341	0.098	-0.133	0.084	-0.112	-0.056	0.081	-0.295	-0.189	-0.021	0.084
Pulse blood oxygen saturation (SPO2)	-0.533	0.3	0.272	0.399	-0.293	-0.032	-0.081	-0.642*	0.187	-0.388	0.028
Body mass index (BMI)	-0.498	0.224	0.497	0.336	-0.364	-0.301	-0.021	-0.098	0.154	0.189	-0.126
White blood cell count (WBC)	-0.078	0.483	0.049	0.608*	-0.455	0.287	-0.007	-0.224	0.476	-0.531	-0.203
Red blood cell count (RBC)	0.438	0.14	-0.105	0.063	-0.329	0	0.448	0.126	0.51	0.175	-0.566
Hemoglobin (Hb)	-0.011	0.483	-0.49	-0.014	-0.333	-0.315	0.578*	0.256	0.298	0.102	-0.364
Platelet count (PLT)	-0.007	-0.014	0.301	0.028	0.021	0.21	-0.35	-0.434	0.336	-0.671*	0
Aspartate amino transferase (AST)	-0.67*	0.286	-0.229	-0.113	0.014	-0.497	0.018	-0.466	-0.236	-0.233	0.212
Total protein (TP)	-0.306	-0.392	0.818*	-0.371	0.322	-0.112	-0.678*	-0.483	-0.168	-0.182	0.273
Albumin (Alb)	0.219	-0.151	0.228	-0.273	0.032	-0.049	-0.375	-0.305	0.032	-0.193	-0.067
Total bilirubin (TBil)	0.064	0.294	-0.182	-0.252	0	0.098	0.21	0.077	0.133	0.133	-0.196
Uric acid (UA)	-0.264	0.203	-0.336	0.088	0.025	0.144	-0.095	0.049	-0.179	-0.333	0.214
Creatinine (CRE)	-0.326	0.269	-0.311	0.106	-0.325	-0.131	0.353	-0.272	0.424	-0.315	-0.258
Fasting blood-glucose (FBG)	0.488	-0.175	0.046	0.011	-0.025	0.245	-0.091	-0.06	0.315	-0.298	-0.249
Pulmonary artery dimension (PAD)	-0.396	0.011	0.014	0.125	0.125	0.136	-0.132	-0.425	-0.411	-0.154	0.439
Left ventricular ejection fraction (LVEF)	0.066	0.458	0.194	0.285	-0.246	0.183	-0.148	0.204	-0.243	0.313	0.046
Right atrium diameter (RAD)	-0.248	-0.474	0.148	-0.357	0.474	-0.216	-0.382	-0.046	-0.421	-0.219	0.445
Right ventricle diameter (RVD)	-0.223	0.186	-0.189	0.133	-0.126	0.105	-0.147	0.06	-0.077	-0.203	0.056
Tricuspid valve max Regurgitation Velocity (m/s) (TVRVmax)	-0.508	-0.406	0.032	-0.399	0.596*	-0.233	-0.353	-0.243	-0.617*	-0.317	0.723*
Pulmonary systolic pressure (PSP)	-0.518	-0.379	0.053	-0.358	0.561	-0.182	-0.365	-0.242	-0.586*	-0.326	0.702*

Supplementary Table 4. The correlation and P-value of clinical parameters and expression of HIF1-α and its target gene VEGFA

Clinical parameters	HIF1A		VEGFA	
	Correlation	Pvalue	Correlation	Pvalue
Age	0.172	0.594	0.042	0.897
Systolic blood pressure (SBP)	-0.525	0.079	-0.252	0.429
Diastolic blood pressure (DBP)	0.095	0.770	-0.081	0.803
Pulse blood oxygen saturation (SPO2)	-0.222	0.488	-0.247	0.439
Body mass index (BMI)	-0.420	0.175	-0.217	0.499
White blood cell count (WBC)	-0.056	0.863	-0.105	0.746
Red blood cell count (RBC)	0.385	0.217	0.720 *	0.008
Hemoglobin (Hb)	0.067	0.837	0.238	0.456
Platelet count (PLT)	0.175	0.587	-0.021	0.948
Aspartate amino transferase (AST)	0.028	0.931	-0.275	0.387
Total protein (TP)	0.035	0.914	-0.140	0.665
Albumin (Alb)	0.483	0.111	0.235	0.463
Total bilirubin (Tobol)	0.210	0.513	0.413	0.183
Uric acid (UA)	0.039	0.905	-0.382	0.221
Creatinine (CRE)	0.081 *	0.035	0.913	0.802
Fasting blood-glucose (FBG)	0.620	0.466	0.127 *	0.032
Pulmonary artery dimension (PAD)	-0.322	0.443	0.149	0.308
Left ventricular ejection fraction (LVEF)	-0.303	0.141	0.662	0.339
Right atrium diameter (RAD)	0.053	0.396	0.203	0.870
Right ventricle diameter (RVD)	0.119	0.273	0.390	0.712
Tricuspid valve max Regurgitation Velocity (m/s) (TVRVmax)	-0.325	0.737	0.006	0.303
Pulmonary systolic pressure (PSP)	-0.326	0.737	0.006	0.301

Supplementary Table 5. The clinical parameters of PH samples

Subjects	P1	P2	P3	P4	P5	P6
Gender (male/female)	Male	Male	Female	Female	Female	Female
Age (years, mean±SD)	53	50	77	66	63	56
Systolic blood pressure (mmHg, mean±SD)	120	149	116	126	124	108
Diastolic blood pressure (mmHg, mean±SD)	80	99	73	94	79	80
Pulse blood oxygen saturation (%, mean±SD)	96	95	99%	83%	70%	88%
Body mass index (kg/m2, mean±SD)	23.00	26.70	28.96	19.50	19.53	19.48
White blood cell count (x10^9/L, mean±SD)	5.44	9.56	5.5	5.47	10.25	5.63
Red blood cell count (x10^{12}/L, mean±SD)	4.84	5.8	3.52	7.01	4.56	4.51
Hemoglobin (g/L, mean±SD)	152	182	120	228	132	157
Platelet count (x10^9/L, mean±SD)	124	195	171	130	208	295
Aspartate amino transferase (U/L, mean±SD)	19.1	21	29	25	16	32
Total protein (g/L, mean±SD)	55.9	67	69	40	80	78
Albumin (g/L, mean±SD)	34.2	40	41	40	36	46
Total bilirubin (μmol/L, mean±SD)	10.1	23.1	15.5	33.1	11	24.2
Uric acid (μmol/L, mean±SD)	344.2	428	425	420	339	500
Creatinine (μmol/L, mean±SD)	68.6	76	175	97	46	91
Fasting blood glucose (mmol/L, mean±SD)	4.21	4.1	9.4	3.2	5	7.4
Pulmonary Artery Dimension (mm, mean±SD)	39	48	32	30	23	30
Left ventricular ejection fraction (%, mean±SD)	77	79	65	85	63	82
Right atrium diameter (mm, mean±SD)	56	56	57	62	48	60
Right ventricle diameter (mm, mean±SD)	44	42	32	35	38	44
TVRVmax (m/s, mean±SD)	4.8	5.2	3.9	4.6	4.4	3.9
Pulmonary systolic pressure (mmHg, mean±SD)	91	112	67	94	80	75

Supplementary Table 6. Primers for real-time PCR

Gene symbol	Species	Forward primer (5′–3′)	Reverse primer (5′–3′)
Aif1	mouse	TCTGCCGTCCAAACTTGAAGC	CCCAAGTTTCTCCAGCATTCG
Fcer1g	mouse	GACTCAAGATCCAGGTCCGAA	TTCAAAGCACAGAGGTGACCA
Tgm2	mouse	TGTACTTCTGGCCCAATCCTGT	AGCAGCCTCAGTTTGTACCCT
Prf1	mouse	TAGCACACGCCTTTAATCCCA	ATAGTAAGCCATTGCAGATCCC
Spon2	mouse	TCATGGCACATCACTTGCT	ATATCCCCAGAGTGTTGCCTA
Tbx21	mouse	CCAACTGAGAAAATGCCGCTGA	GCAGAAAGCCATGAAGTCCCT
Clec1b	mouse	AAGGATTACTTCAGTCCGTTG	TTTCCATTATGAAGATAAGCACA
Nfe2	mouse	CAGCCTGGTCTACATAGCAAG	AAGTTGCCTTCTCTTACTCCC
*Clec4a**	mouse	ATGTGCTACAATAATTTACCG	ATGAGTAAACAAGATGGCAA
Tlr2	mouse	TGACCCGCCCTTTAAGCTG	GTTCGTACTTGCACCACTCG
Cd28	mouse	TCCTCTGAACACAACCGAA	ATTAGAAATTACAGGCACACC
Cd3e	mouse	TCAGAAGCATGATAAGCACCT	CAAGCCCAGAGTGATACAGA
Lag3	mouse	ACCCCTTCTTTGCTCATTGCC	AGACCCACAGCCTCAAGGTG
Gp6	mouse	CCAATTTCCCCATCATCACA	GCAGTGATATTCATAGGCTTT
Vwf	mouse	ACATCCTCACATACACGCCACA	TGACCGTGCCATCTCGCAAC
Actb	mouse	GTGACGTTGACATCCGTAAAGA	GCCGGACTCATCGTACTCC

* *Clec4a* is expressed only in human and rats, which corresponds to *Clec4a2* in mice.

Supplementary Table 7. siRNA sequences for *CD28*, *SPON2* and *TBX21*

Gene symbol	Species		Sequences (5′–3′)
CD28	human	siRNA-1	GCUGCAAGUAUUCCUACAATT
	human	siRNA-2	CUCCUUACCUAGACAAUGATT
SPON2	human	siRNA-1	GGGACAAUGAGAUUGUAGATT
	human	siRNA-2	GGACACGGUGACCGAGAUATT
TBX21	human	siRNA-1	GCUUCCAACACGCAUAUCUTT
	human	siRNA-2	GCGUGAGGACUACGCGCUATT

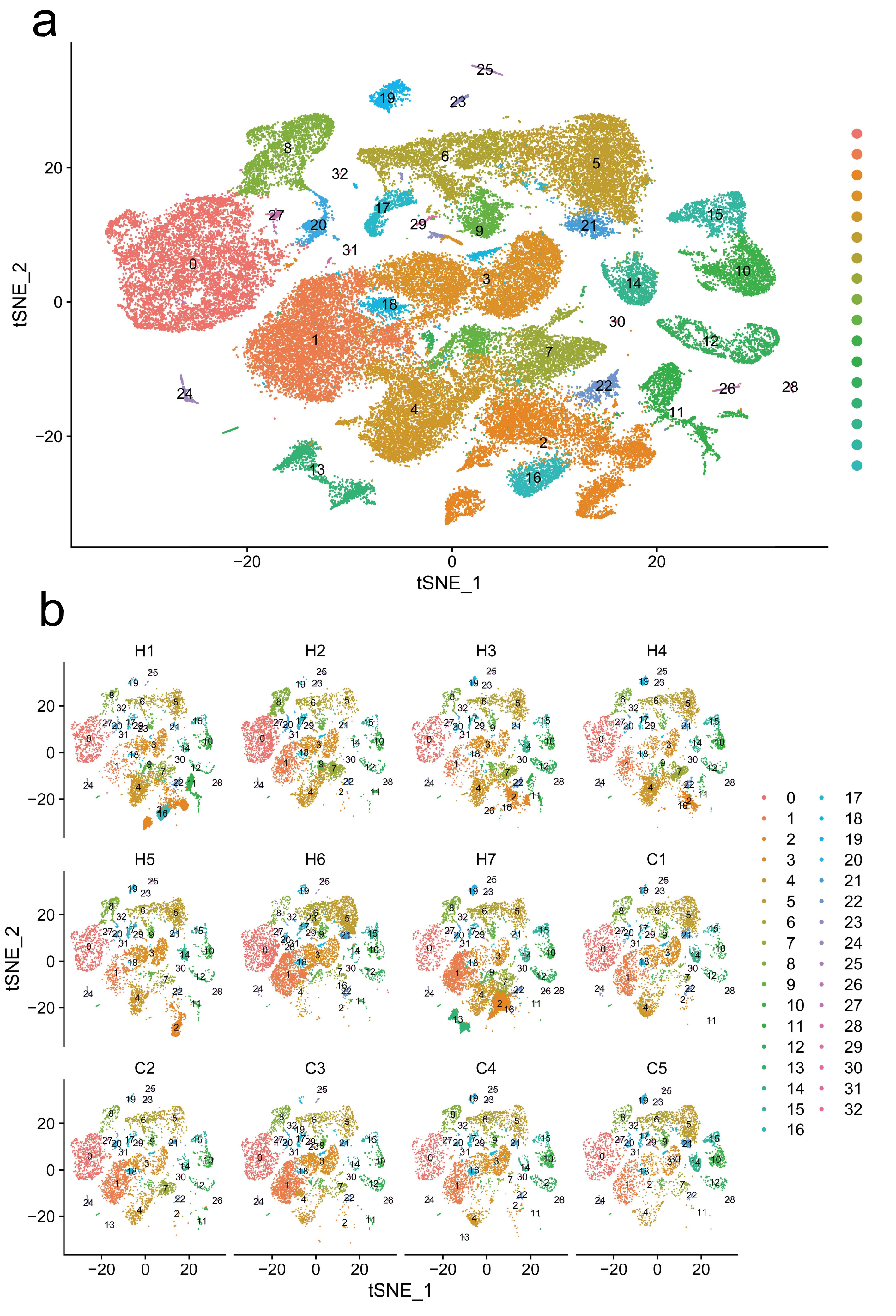

Supplementary Figure 1. The immune landscape of PBMC samples from patients with HAPH.

Supplementary Figure 1. The immune landscape of PBMC samples from patients with HAPH. (a) t-SNE (t-distributed stochastic neighbor embedding) plot of all cells that passed quality control. **(b)** t-SNE plot of all cells that passed quality control in each individual subject. Cells were colored based on 31 clusters defined by k-means clustering.

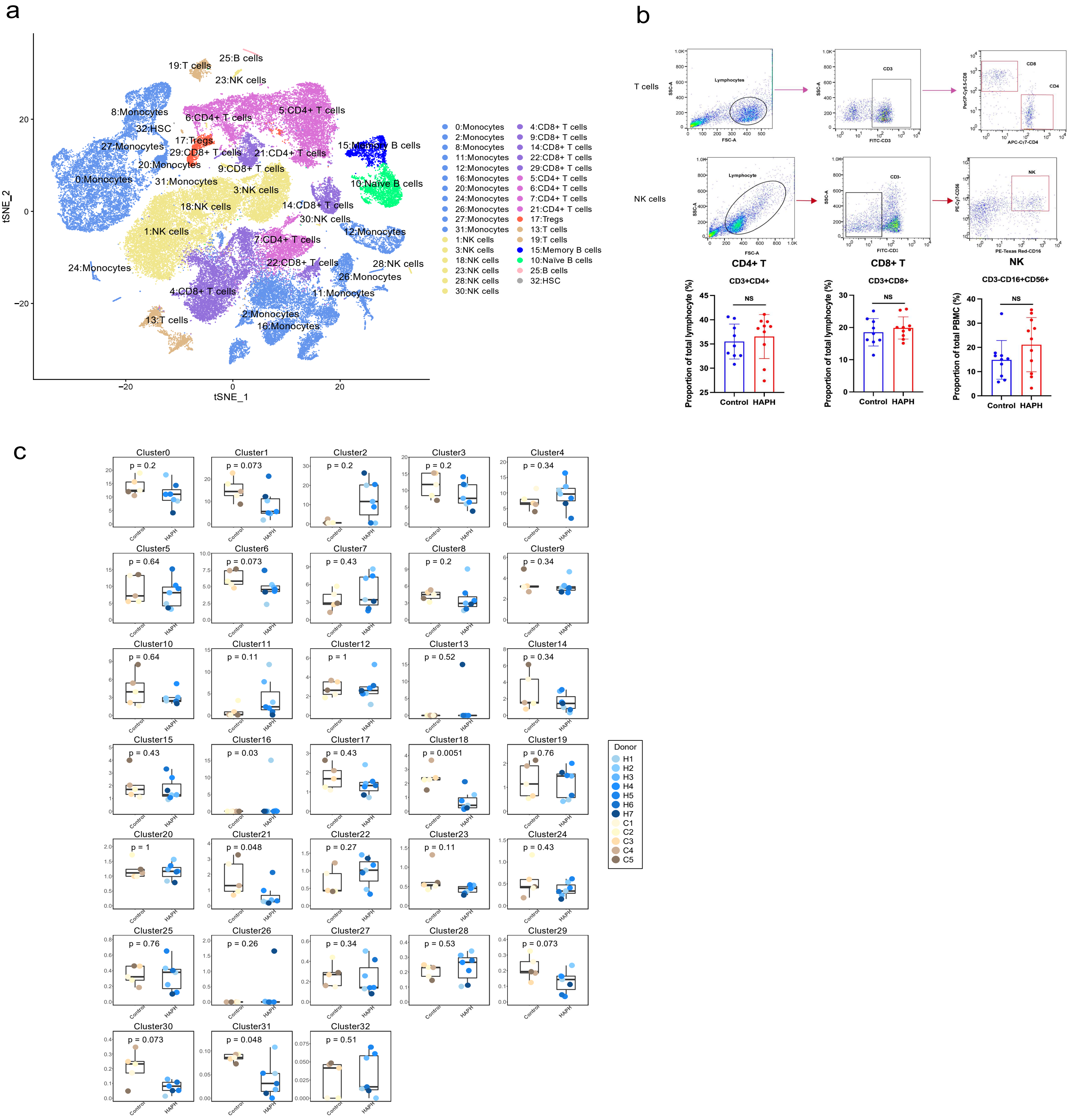

Supplementary Figure 2. The immune landscape of PBMC samples from patients with HAPH.

Supplementary Figure 2. The immune landscape of PBMC samples from patients with HAPH. (a) The entire dataset colored by orthogonally generated clusters labeled by manual cell type annotation. **(b)** FACS validation of main immune cell populations ($*p < 0.05$; Unpaired t test was utilized as appropriate. Data were presented as mean ± SD). **(c)** Proportions of each cluster in each sample. Two-sided *p* values by the Wilcoxon rank-sum test.

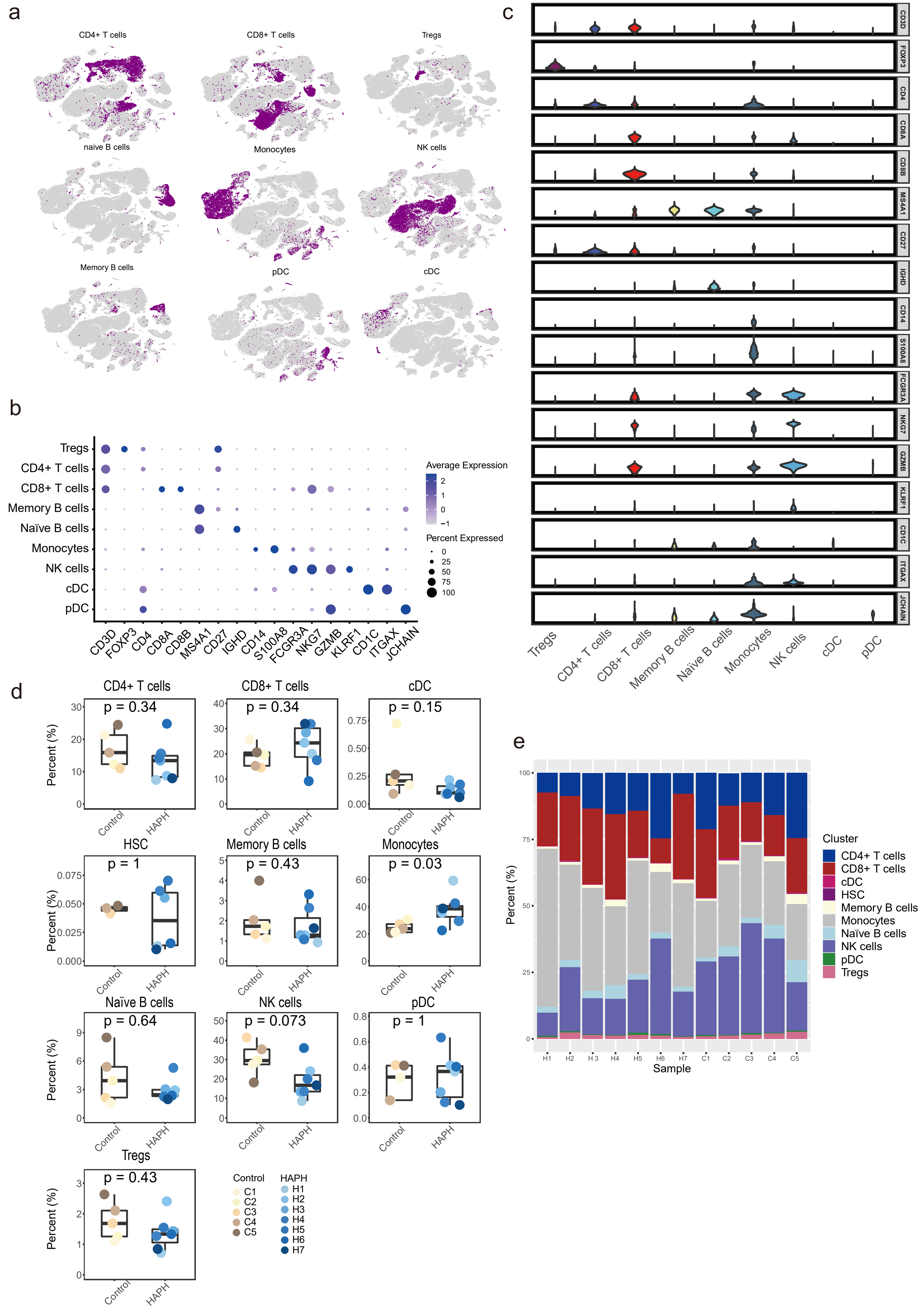

Supplementary Figure 3. Differential immune cell composition in the periphery of patients with HAPH.

Supplementary Figure 3. Differential immune cell composition in the periphery of patients with HAPH. (**a**) tSNE (t-distributed stochastic neighbor embedding) plot of the main immune cell subsets. (**b**) Dot plot depicting the percentages and average expressions of the canonical genes associated with each main immune cell cluster. (**c**) Violin plots indicating the gene expressions in each main cluster (cluster numbers as in Figure 2a) of cells from both the HAPH and control PBMC samples. (**d**) Boxplots comparing the percentages of each main cell type between the HAPH (n=7) and control (n=5) PBMC samples. The x axes correspond to each group. The two-sided *p* values from the Wilcoxon rank-sum test are shown. (**e**) Proportions of each cell type in each sample.

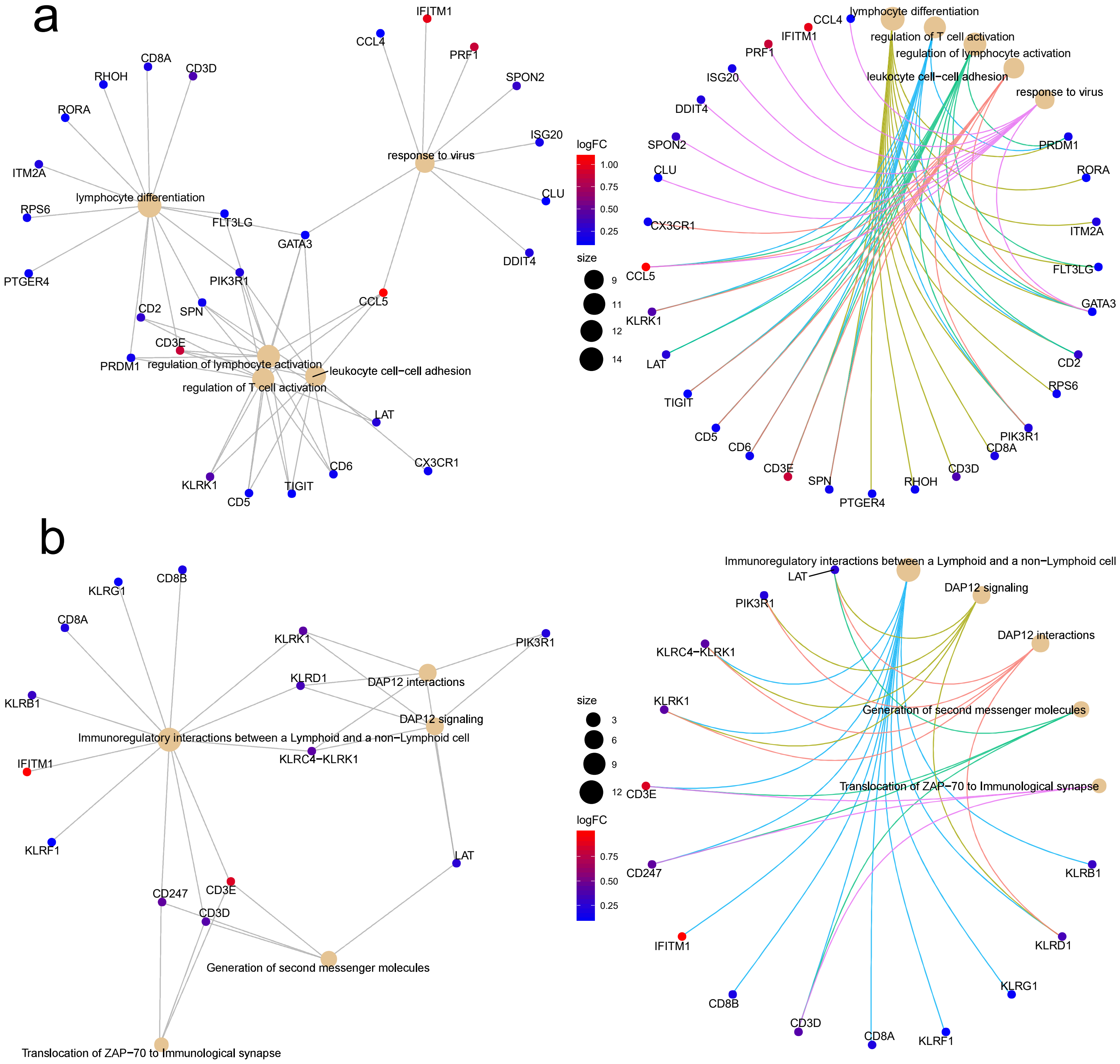

Supplementary Figure 4. Differentially expressed genes (DEGs) in cluster 0 (C0) monocyte between HAPH patients and controls.

Supplementary Figure 4. Differentially expressed genes (DEGs) in cluster 0 (C0) monocyte between HAPH patients and controls. (a) Network visualization of DEGs by Gene Ontology analysis (biological process). **(b)** Network visualization of DEGs by pathway enrichment analysis (Reactome database).

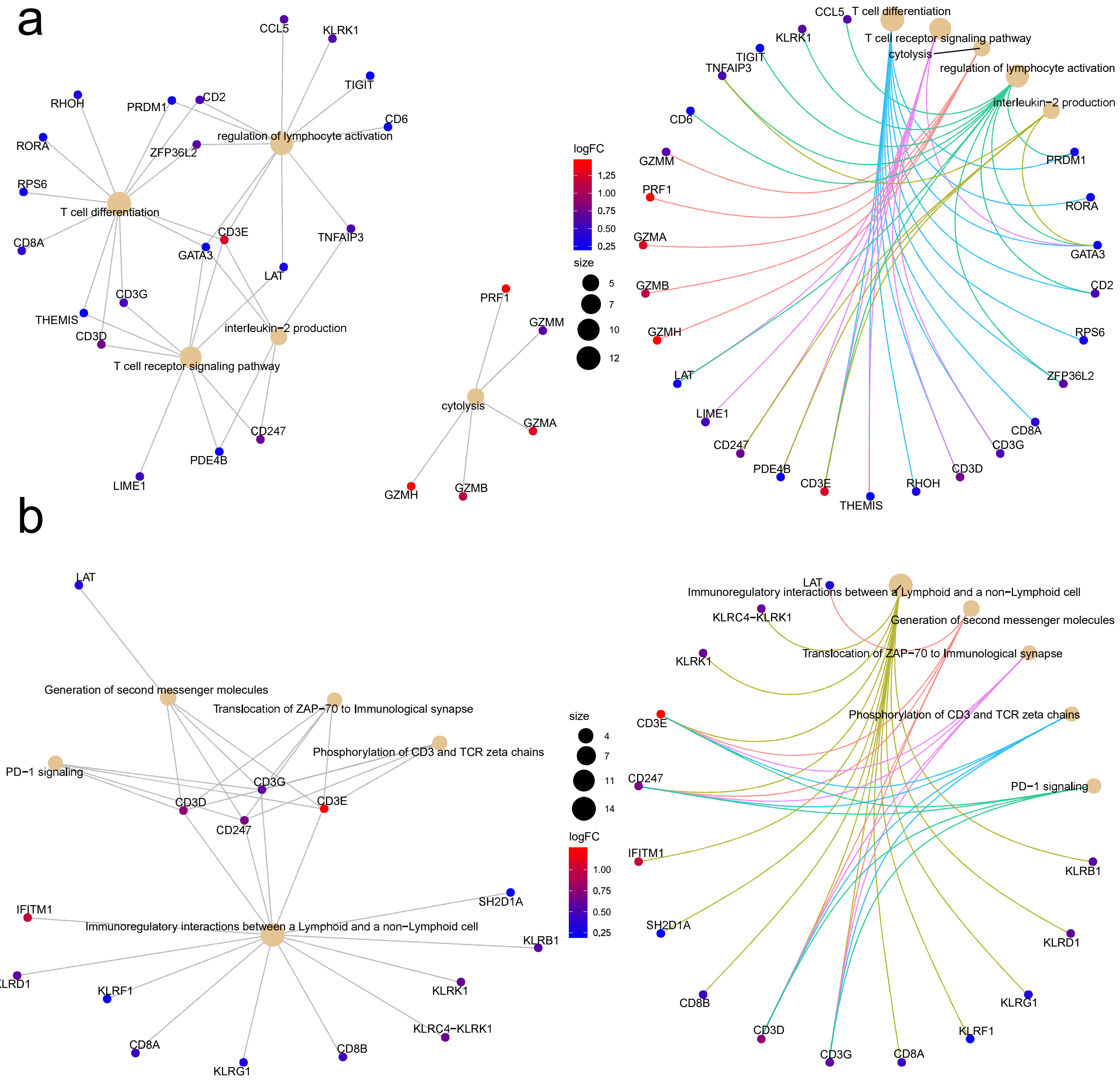

Supplementary Figure 5. Differentially expressed genes (DEGs) in cluster 1 (C1) monocyte between HAPH patients and controls.

Supplementary Figure 5. Differentially expressed genes (DEGs) in cluster 1 (C1) monocyte between HAPH patients and controls. (a) Network visualization of DEGs by Gene Ontology analysis (biological process). **(b)** Network visualization of DEGs by pathway enrichment analysis (Reactome database).

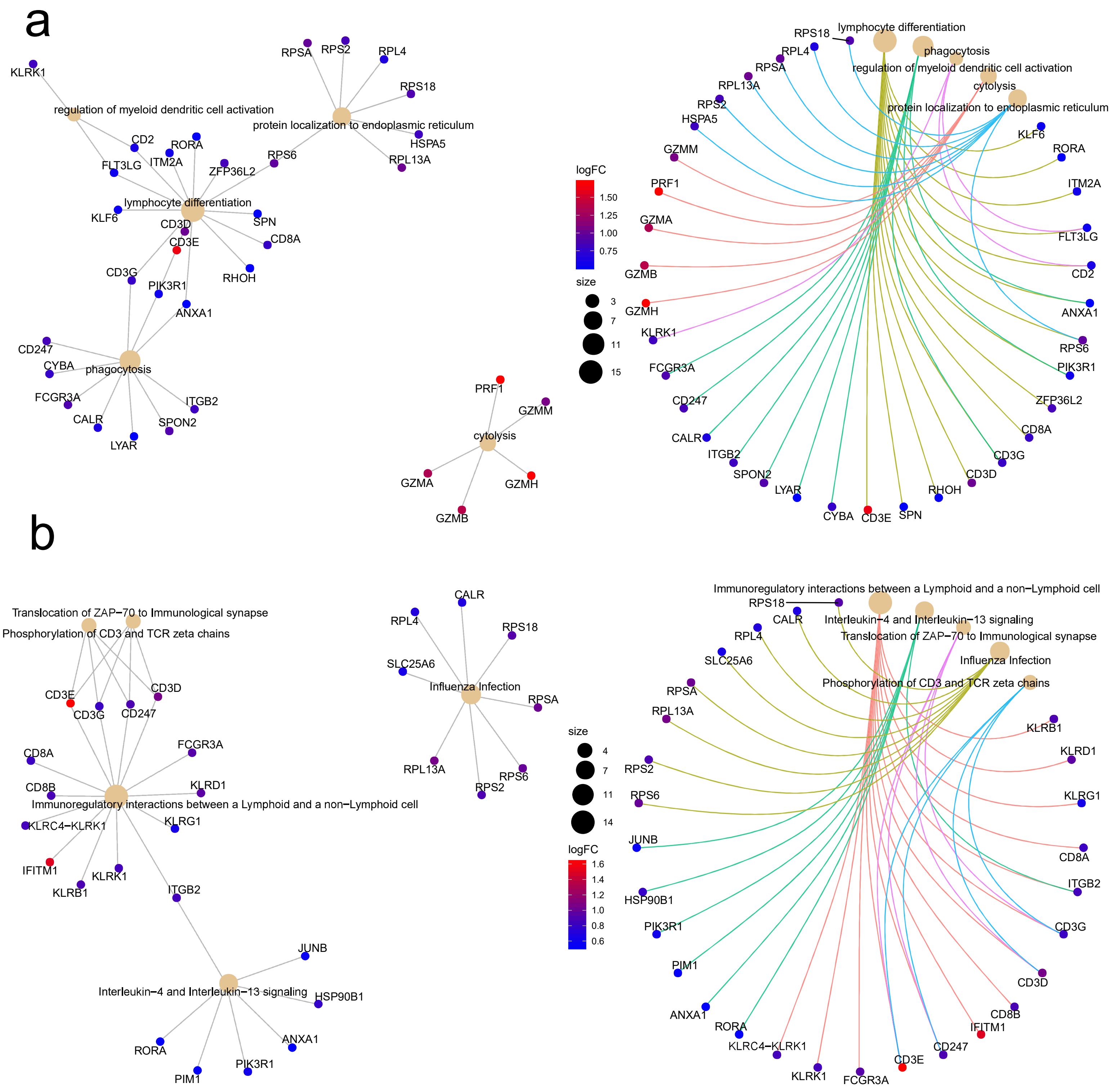

Supplementary Figure 6. Differentially expressed genes (DEGs) in cluster 2 (C2) monocyte between HAPH patients and controls.

Supplementary Figure 6. Differentially expressed genes (DEGs) in cluster 2 (C2) monocyte between HAPH patients and controls. (a) Network visualization of DEGs by Gene Ontology analysis (biological process). **(b)** Network visualization of DEGs by pathway enrichment analysis (Reactome database).

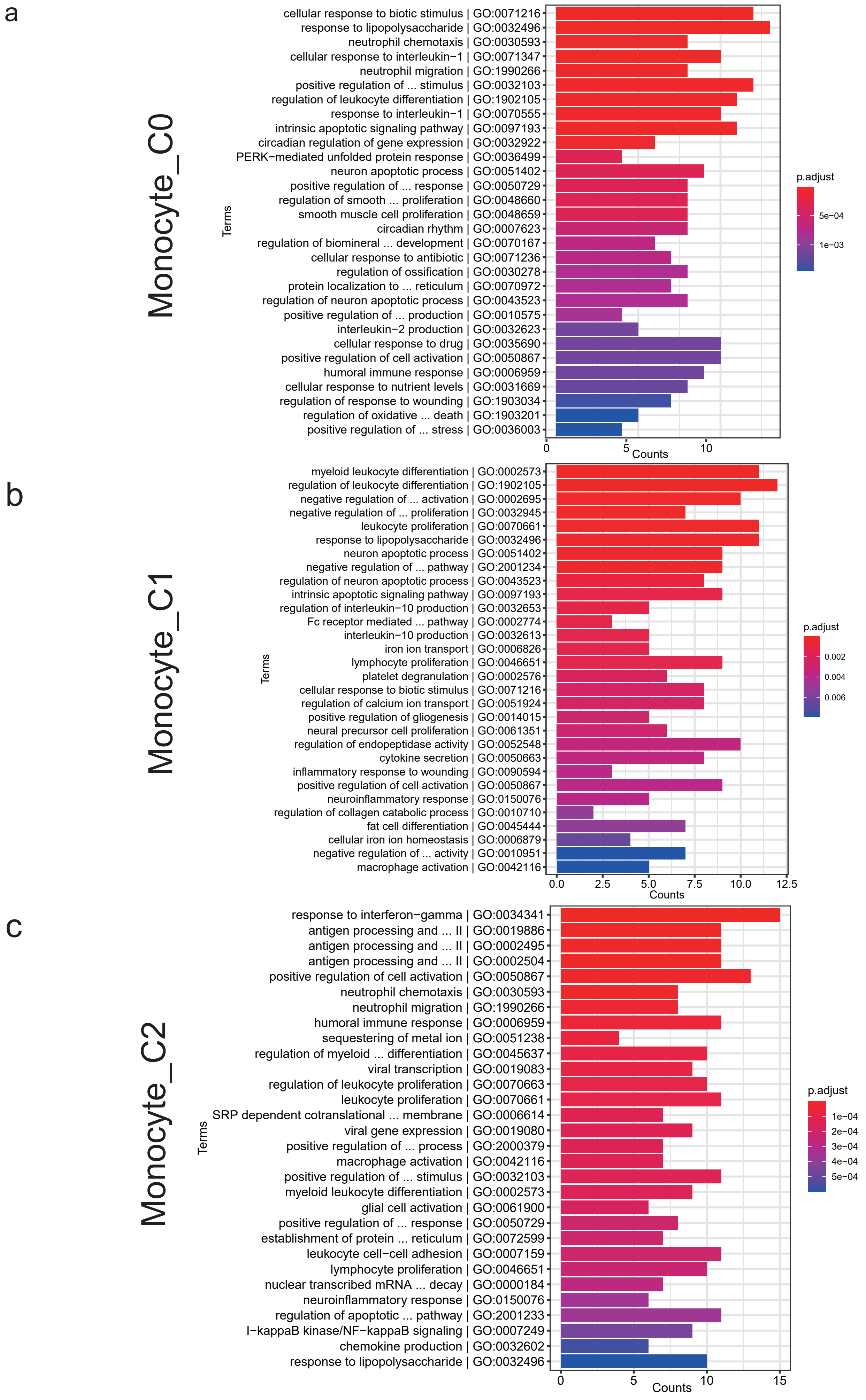

Supplementary Figure 7. Gene Ontology analysis (biological process) of the down-regulated genes in each monocyte subsets.

Supplementary Figure 7. Gene Ontology analysis (biological process) of the down-regulated genes in monocyte C0 (**a**), monocyte C1 (**b**) and monocyte C2 (**c**).

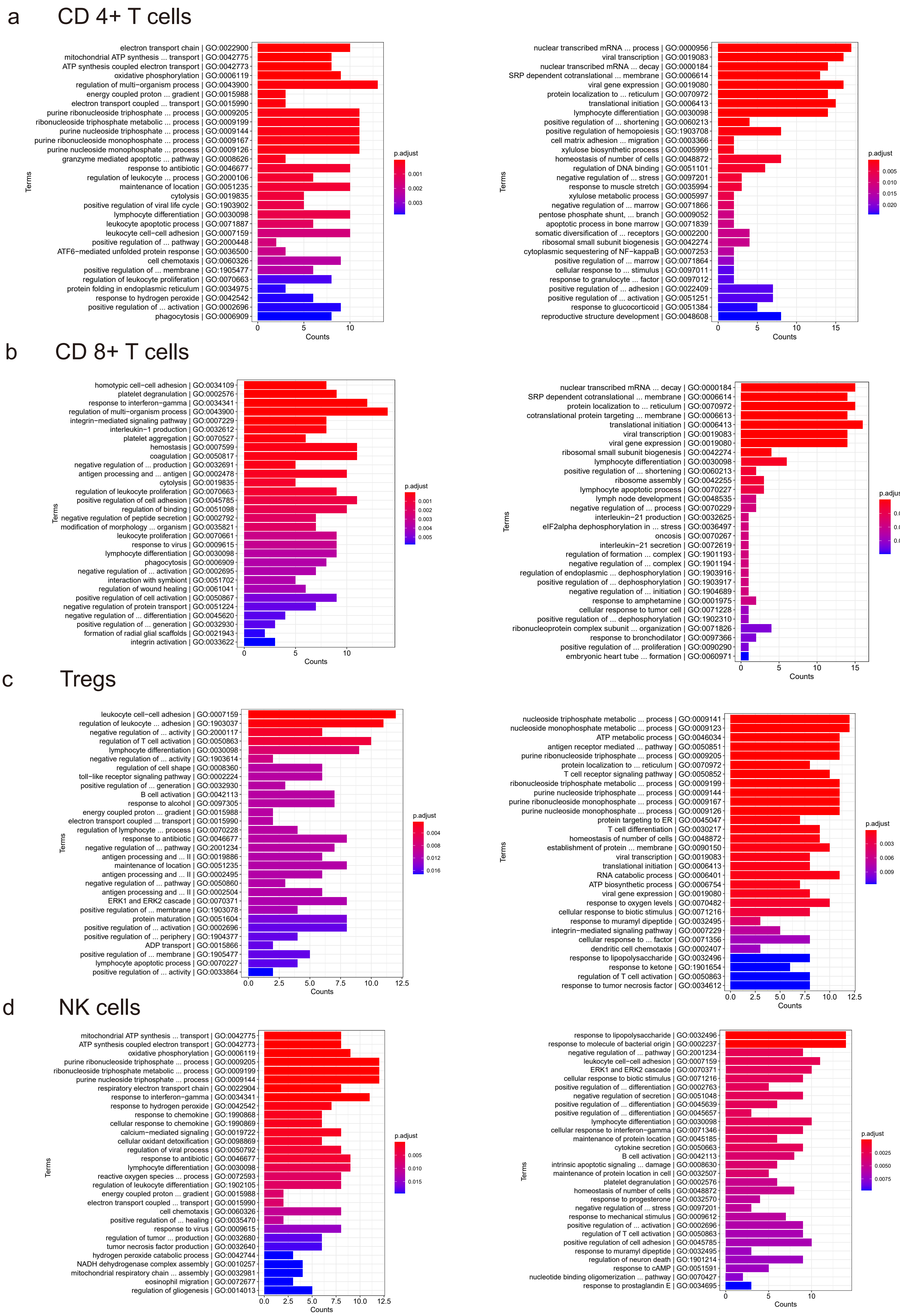
a
CD 4+ T cells
electron transport chain | GO:0022900
mitochondrial ATP synthesis ... transport | GO:0042775
ATP synthesis coupled electron transport | GO:0042773
oxidative phosphorylation | GO:0006119
regulation of multi−organism process | GO:0043900
energy coupled proton ... gradient | GO:0015988
electron transport coupled ... transport | GO:0015990
purine ribonucleoside triphosphate ... process | GO:0009205
ribonucleoside triphosphate metabolic ... process | GO:0009199
purine nucleoside triphosphate ... process | GO:0009144
purine ribonucleoside monophosphate ... process | GO:0009167
purine nucleoside monophosphate ... process | GO:0009126
granzyme mediated apoptotic ... pathway | GO:0008626
response to antibiotic | GO:0046677
regulation of leukocyte ... process | GO:2000106
maintenance of location | GO:0051235
cytolysis | GO:0019835
positive regulation of viral life cycle | GO:1903902
lymphocyte differentiation | GO:0030098
leukocyte apoptotic process | GO:0071887
leukocyte cell−cell adhesion | GO:0007159
positive regulation of ... pathway | GO:2000448
ATF6−mediated unfolded protein response | GO:0036500
cell chemotaxis | GO:0060326
positive regulation of ... membrane | GO:1905477
regulation of leukocyte proliferation | GO:0070663
protein folding in endoplasmic reticulum | GO:0034975
response to hydrogen peroxide | GO:0042542
positive regulation of ... activation | GO:0002696
phagocytosis | GO:0006909
Terms
Counts
0
5
10
p.adjust
0.001
0.002
0.003
nuclear transcribed mRNA ... process | GO:0000956
viral transcription | GO:0019083
nuclear transcribed mRNA ... decay | GO:0000184
SRP dependent cotranslational ... membrane | GO:0006614
viral gene expression | GO:0019080
protein localization to ... reticulum | GO:0070972
translational initiation | GO:0006413
lymphocyte differentiation | GO:0030098
positive regulation of ... shortening | GO:0060213
positive regulation of hemopoiesis | GO:1903708
cell matrix adhesion ... migration | GO:0003366
xylulose biosynthetic process | GO:0005999
homeostasis of number of cells | GO:0048872
regulation of DNA binding | GO:0051101
negative regulation of ... stress | GO:0097201
response to muscle stretch | GO:0035994
xylulose metabolic process | GO:0005997
negative regulation of ... marrow | GO:0071866
pentose phosphate shunt, ... branch | GO:0009052
apoptotic process in bone marrow | GO:0071839
somatic diversification of ... receptors | GO:0002200
ribosomal small subunit biogenesis | GO:0042274
cytoplasmic sequestering of NF−kappaB | GO:0007253
positive regulation of ... marrow | GO:0071864
cellular response to ... stimulus | GO:0097011
response to granulocyte ... factor | GO:0097012
positive regulation of ... adhesion | GO:0022409
positive regulation of ... activation | GO:0051251
response to glucocorticoid | GO:0051384
reproductive structure development | GO:0048608
Terms
Counts
0
5
10
15
p.adjust
0.005
0.010
0.015
0.020
b
CD 8+ T cells
homotypic cell−cell adhesion | GO:0034109
platelet degranulation | GO:0002576
response to interferon−gamma | GO:0034341
regulation of multi−organism process | GO:0043900
integrin−mediated signaling pathway | GO:0007229
interleukin−1 production | GO:0032612
platelet aggregation | GO:0070527
hemostasis | GO:0007599
coagulation | GO:0050817
negative regulation of ... production | GO:0032691
antigen processing and ... antigen | GO:0002478
cytolysis | GO:0019835
regulation of leukocyte proliferation | GO:0070663
positive regulation of cell adhesion | GO:0045785
regulation of binding | GO:0051098
negative regulation of peptide secretion | GO:0002792
modification of morphology ... organism | GO:0035821
leukocyte proliferation | GO:0070661
response to virus | GO:0009615
lymphocyte differentiation | GO:0030098
phagocytosis | GO:0006909
negative regulation of ... activation | GO:0002695
interaction with symbiont | GO:0051702
regulation of wound healing | GO:0061041
positive regulation of cell activation | GO:0050867
negative regulation of protein transport | GO:0051224
negative regulation of ... differentiation | GO:0045620
positive regulation of ... generation | GO:0032930
formation of radial glial scaffolds | GO:0021943
integrin activation | GO:0033622
Terms
Counts
0
5
10
p.adjust
0.001
0.002
0.003
0.004
0.005
nuclear transcribed mRNA ... decay | GO:0000184
SRP dependent cotranslational ... membrane | GO:0006614
protein localization to ... reticulum | GO:0070972
cotranslational protein targeting ... membrane | GO:0006613
translational initiation | GO:0006413
viral transcription | GO:0019083
viral gene expression | GO:0019080
ribosomal small subunit biogenesis | GO:0042274
lymphocyte differentiation | GO:0030098
positive regulation of ... shortening | GO:0060213
ribosome assembly | GO:0042255
lymphocyte apoptotic process | GO:0070227
lymph node development | GO:0048535
negative regulation of ... process | GO:0070229
interleukin−21 production | GO:0032625
eIF2alpha dephosphorylation in ... stress | GO:0036497
oncosis | GO:0070267
interleukin−21 secretion | GO:0072619
regulation of formation ... complex | GO:1901193
negative regulation of ... complex | GO:1901194
regulation of endoplasmic ... dephosphorylation | GO:1903916
positive regulation of ... dephosphorylation | GO:1903917
negative regulation of ... initiation | GO:1904689
response to amphetamine | GO:0001975
cellular response to tumor cell | GO:0071228
positive regulation of ... dephosphorylation | GO:1902310
ribonucleoprotein complex subunit ... organization | GO:0071826
response to bronchodilator | GO:0097366
positive regulation of ... proliferation | GO:0090290
embryonic heart tube ... formation | GO:0060971
Terms
Counts
0
5
10
15
p.adjust
0.025
0.050
0.075
c
Tregs
leukocyte cell−cell adhesion | GO:0007159
regulation of leukocyte ... adhesion | GO:1903037
negative regulation of ... activity | GO:2000117
regulation of T cell activation | GO:0050863
lymphocyte differentiation | GO:0030098
negative regulation of ... activity | GO:1903614
regulation of cell shape | GO:0008360
toll−like receptor signaling pathway | GO:0002224
positive regulation of ... generation | GO:0032930
B cell activation | GO:0042113
response to alcohol | GO:0097305
energy coupled proton ... gradient | GO:0015988
electron transport coupled ... transport | GO:0015990
regulation of lymphocyte ... process | GO:0070228
response to antibiotic | GO:0046677
negative regulation of ... pathway | GO:2001234
antigen processing and ... II | GO:0019886
maintenance of location | GO:0051235
antigen processing and ... II | GO:0002495
negative regulation of ... pathway | GO:0050860
antigen processing and ... II | GO:0002504
ERK1 and ERK2 cascade | GO:0070371
positive regulation of ... membrane | GO:1903078
protein maturation | GO:0051604
positive regulation of ... activation | GO:0002696
positive regulation of ... periphery | GO:1904377
ADP transport | GO:0015866
positive regulation of ... membrane | GO:1905477
lymphocyte apoptotic process | GO:0070227
positive regulation of ... activity | GO:0033864
Terms
Counts
0.0
2.5
5.0
7.5
10.0
12.5
p.adjust
0.004
0.008
0.012
0.016
nucleoside triphosphate metabolic ... process | GO:0009141
nucleoside monophosphate metabolic ... process | GO:0009123
ATP metabolic process | GO:0046034
antigen receptor mediated ... pathway | GO:0050851
purine ribonucleoside triphosphate ... process | GO:0009205
protein localization to ... reticulum | GO:0070972
T cell receptor signaling pathway | GO:0050852
ribonucleoside triphosphate metabolic ... process | GO:0009199
purine nucleoside triphosphate ... process | GO:0009144
purine ribonucleoside monophosphate ... process | GO:0009167
purine nucleoside monophosphate ... process | GO:0009126
protein targeting to ER | GO:0045047
T cell differentiation | GO:0030217
homeostasis of number of cells | GO:0048872
establishment of protein ... membrane | GO:0090150
viral transcription | GO:0019083
translational initiation | GO:0006413
RNA catabolic process | GO:0006401
ATP biosynthetic process | GO:0006754
viral gene expression | GO:0019080
response to oxygen levels | GO:0070482
cellular response to biotic stimulus | GO:0071216
response to muramyl dipeptide | GO:0032495
integrin−mediated signaling pathway | GO:0007229
cellular response to ... factor | GO:0071356
dendritic cell chemotaxis | GO:0002407
response to lipopolysaccharide | GO:0032496
response to ketone | GO:1901654
regulation of T cell activation | GO:0050863
response to tumor necrosis factor | GO:0034612
Terms
Counts
0.0
2.5
5.0
7.5
10.0
12.5
p.adjust
0.003
0.006
0.009
d
NK cells
mitochondrial ATP synthesis ... transport | GO:0042775
ATP synthesis coupled electron transport | GO:0042773
oxidative phosphorylation | GO:0006119
purine ribonucleoside triphosphate ... process | GO:0009205
ribonucleoside triphosphate metabolic ... process | GO:0009199
purine nucleoside triphosphate ... process | GO:0009144
respiratory electron transport chain | GO:0022904
response to interferon−gamma | GO:0034341
response to hydrogen peroxide | GO:0042542
response to chemokine | GO:1990868
cellular response to chemokine | GO:1990869
calcium−mediated signaling | GO:0019722
cellular oxidant detoxification | GO:0098869
regulation of viral process | GO:0050792
response to antibiotic | GO:0046677
lymphocyte differentiation | GO:0030098
reactive oxygen species ... process | GO:0072593
regulation of leukocyte differentiation | GO:1902105
energy coupled proton ... gradient | GO:0015988
electron transport coupled ... transport | GO:0015990
cell chemotaxis | GO:0060326
positive regulation of ... healing | GO:0035470
response to virus | GO:0009615
regulation of tumor ... production | GO:0032680
tumor necrosis factor production | GO:0032640
hydrogen peroxide catabolic process | GO:0042744
NADH dehydrogenase complex assembly | GO:0010257
mitochondrial respiratory chain ... assembly | GO:0032981
eosinophil migration | GO:0072677
regulation of gliogenesis | GO:0014013
Terms
Counts
0.0
2.5
5.0
7.5
10.0
12.5
p.adjust
0.005
0.010
0.015
response to lipopolysaccharide | GO:0032496
response to molecule of bacterial origin | GO:0002237
negative regulation of ... pathway | GO:2001234
leukocyte cell−cell adhesion | GO:0007159
ERK1 and ERK2 cascade | GO:0070371
cellular response to biotic stimulus | GO:0071216
positive regulation of ... differentiation | GO:0002763
negative regulation of secretion | GO:0051048
positive regulation of ... differentiation | GO:0045639
positive regulation of ... differentiation | GO:0045657
lymphocyte differentiation | GO:0030098
cellular response to interferon−gamma | GO:0071346
maintenance of protein location | GO:0045185
cytokine secretion | GO:0050663
B cell activation | GO:0042113
intrinsic apoptotic signaling ... damage | GO:0008630
maintenance of location in cell | GO:0032507
platelet degranulation | GO:0002576
homeostasis of number of cells | GO:0048872
response to progesterone | GO:0032570
negative regulation of ... stress | GO:0097201
response to mechanical stimulus | GO:0009612
positive regulation of ... activation | GO:0002696
regulation of T cell activation | GO:0050863
positive regulation of cell adhesion | GO:0045785
response to muramyl dipeptide | GO:0032495
regulation of neuron death | GO:1901214
response to cAMP | GO:0051591
nucleotide binding oligomerization ... pathway | GO:0070427
response to prostaglandin E | GO:0034695
Terms
Counts
0
5
10
p.adjust
0.0025
0.0050
0.0075

e memory B cells

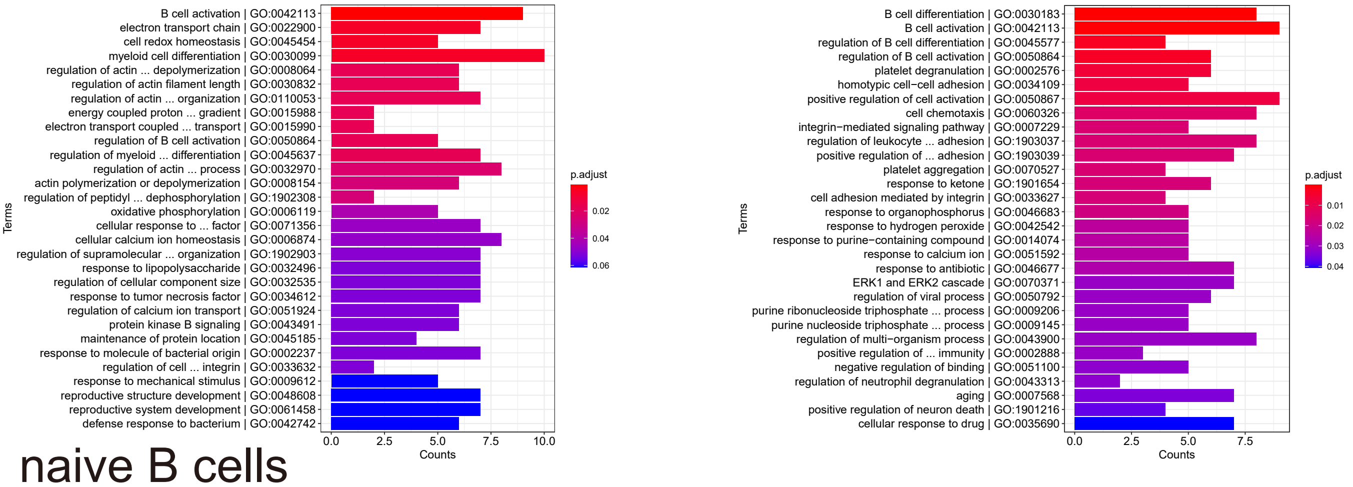

f naive B cells

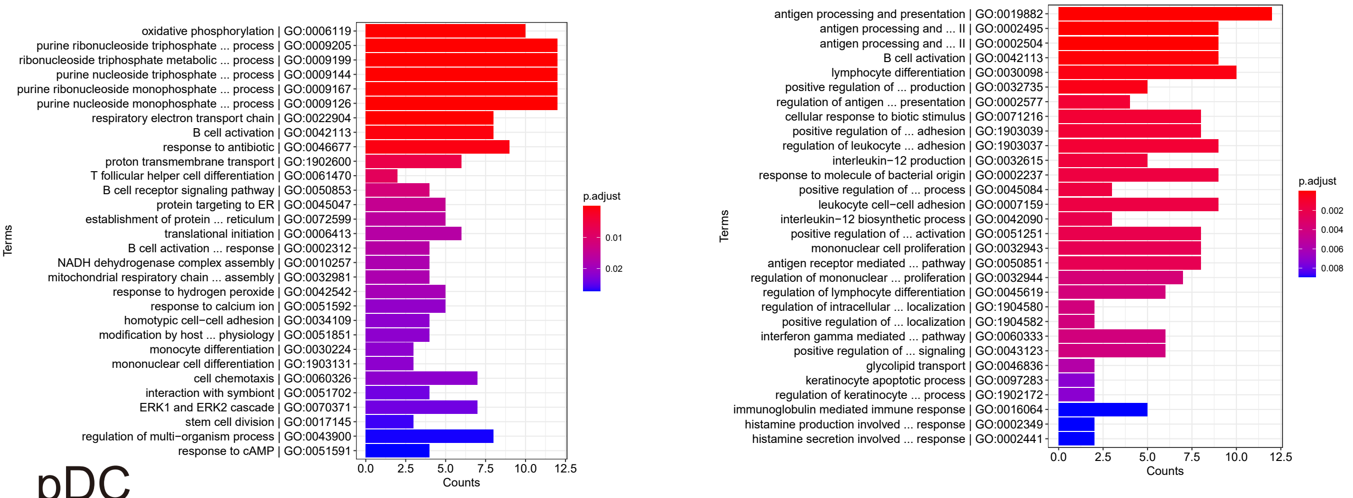

g pDC

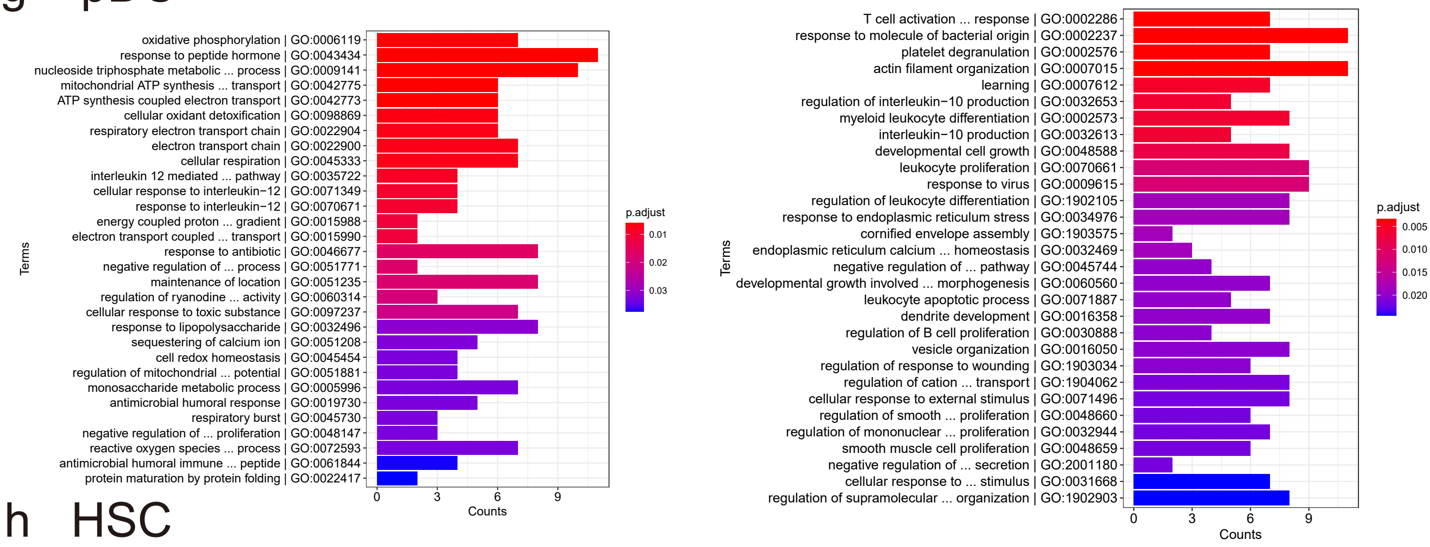

h HSC

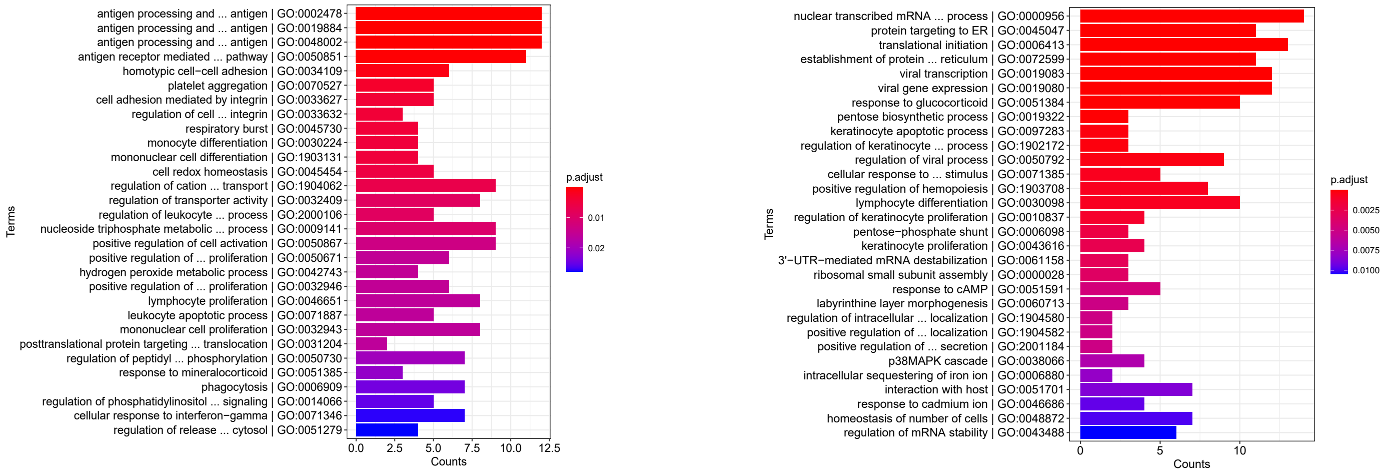

Supplementary Figure 8. Gene Ontology analysis (biological process) of the up-regulated and down-regulated genes in each cell type in comparison of HAPH with control group.

Supplementary Figure 8. Gene Ontology analysis (biological process) in $CD4^+$ T cells **(a)**, $CD8^+$ T cells **(b)**, Tregs **(c)**, NK cells **(d)**, Memory B cells **(e)**, Naive B cells **(f)**, pDC **(g)**, HSC **(h)** in comparison of HAPH with control group (up-regulated genes on left panel and down-regulated genes on right panel).

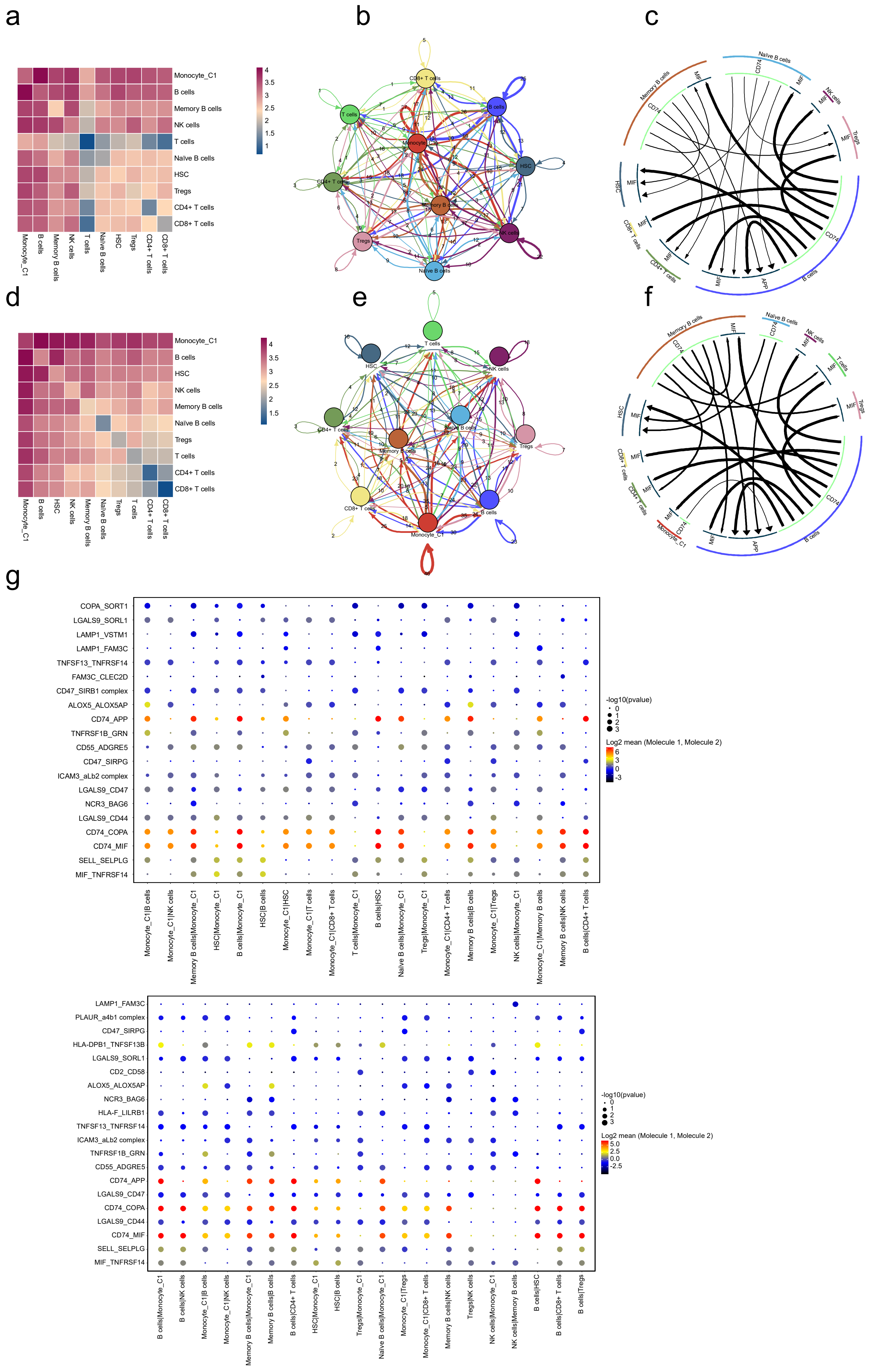

Supplementary Figure 9. Cell–cell communication networks between C1 monocyte and other peripheral blood cell types using CellPhoneDB.

Supplementary Figure 9. Cell–cell communication networks between C1 monocyte and other peripheral blood cell types using CellPhoneDB in HAPH patients (**a-c**) and controls (**d-f**). Top 20 ligand-receptor pairs were listed in HAPH and control group (**g**).

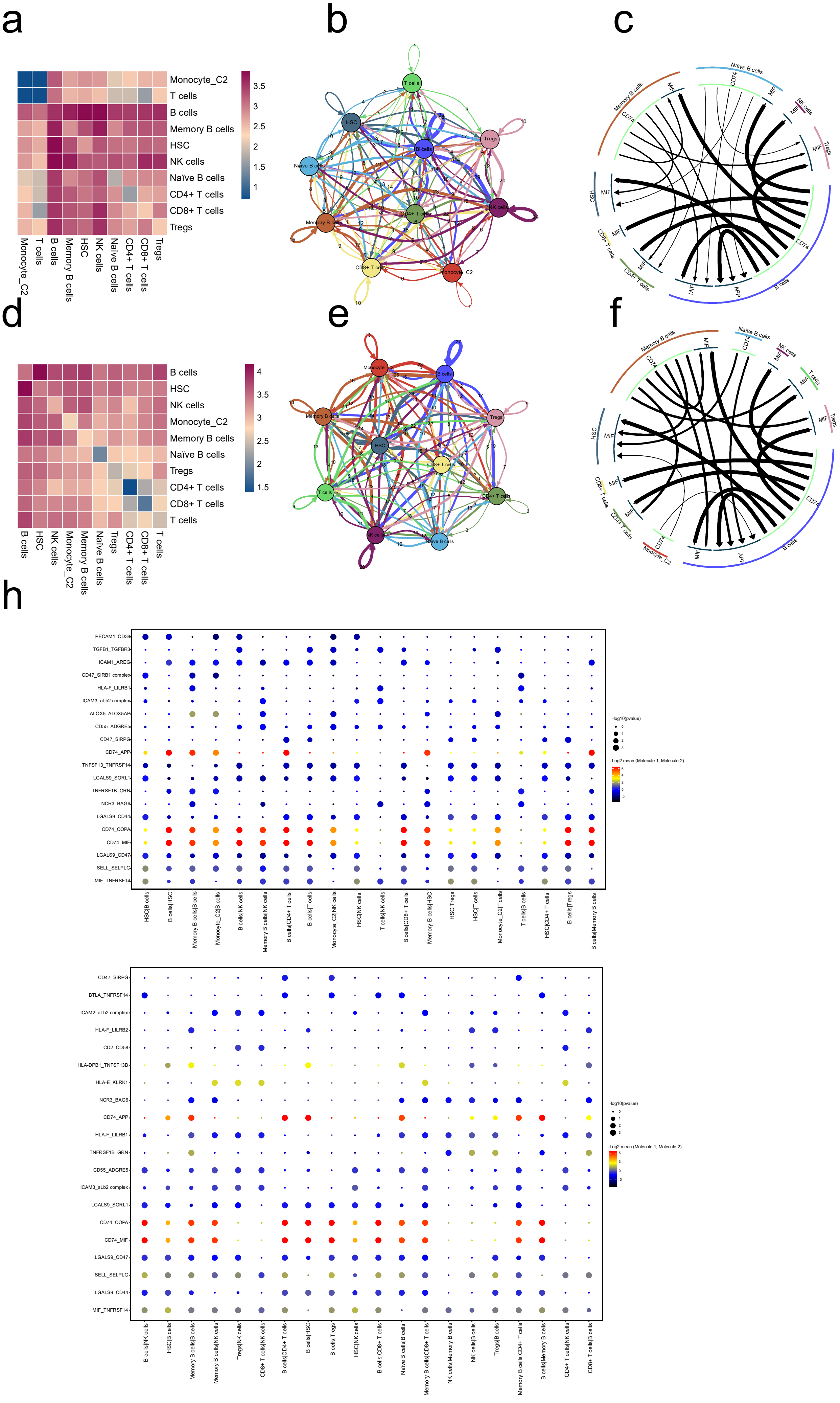

Supplementary Figure 10. Cell–cell communication networks between C2 monocyte and other peripheral blood cell types using CellPhoneDB.

Supplementary Figure 10. Cell–cell communication networks between C2 monocyte and other peripheral blood cell types using CellPhoneDB in HAPH (**a-c**) and control (**d-f**) individuals. Top 20 ligand-receptor pairs in HAPH and control group (**g**).

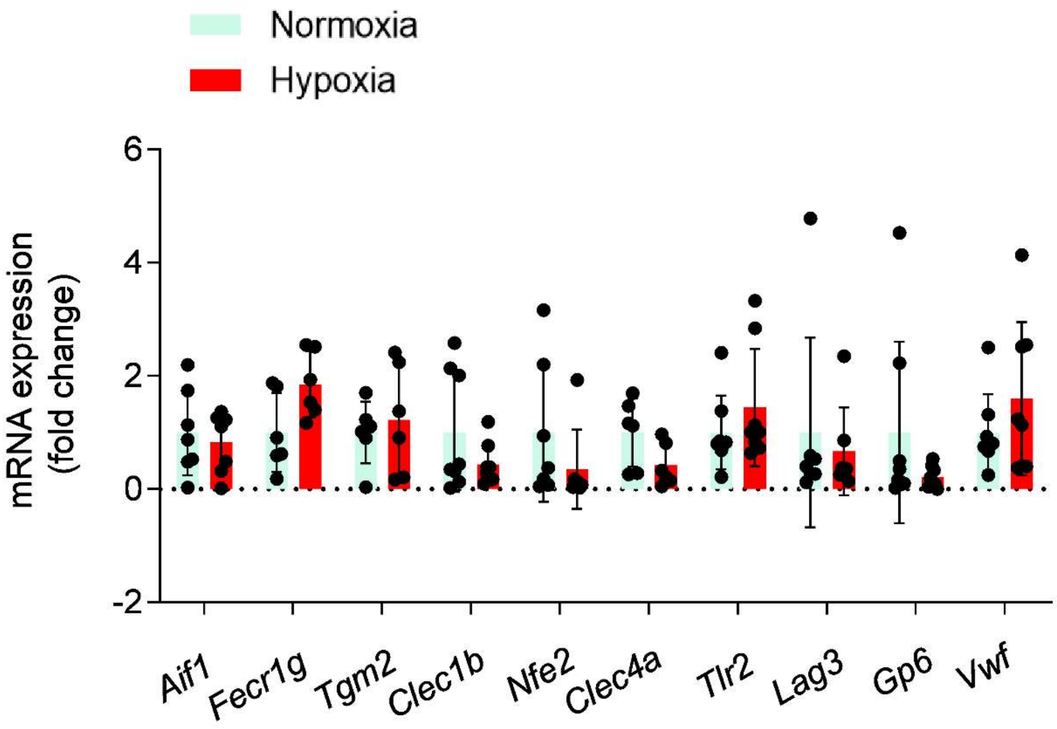

Figure S11

Supplementary Figure 11. The relative mRNA expression of *Aif1*, *Fecr1g*, *Tgm2*, *Clec1b*, *Nfe2*, *Clec4a*, *Tlr2*, *Lag3*, *Gp6* and *Vwf* in lung tissues of the mice exposed to normoxia (21% O_2) or hypoxia (10% O_2) for 4 weeks (n = 6-8 for per group, *$p < 0.05$; Student's *t*-test. Data were presented as mean ± SD).

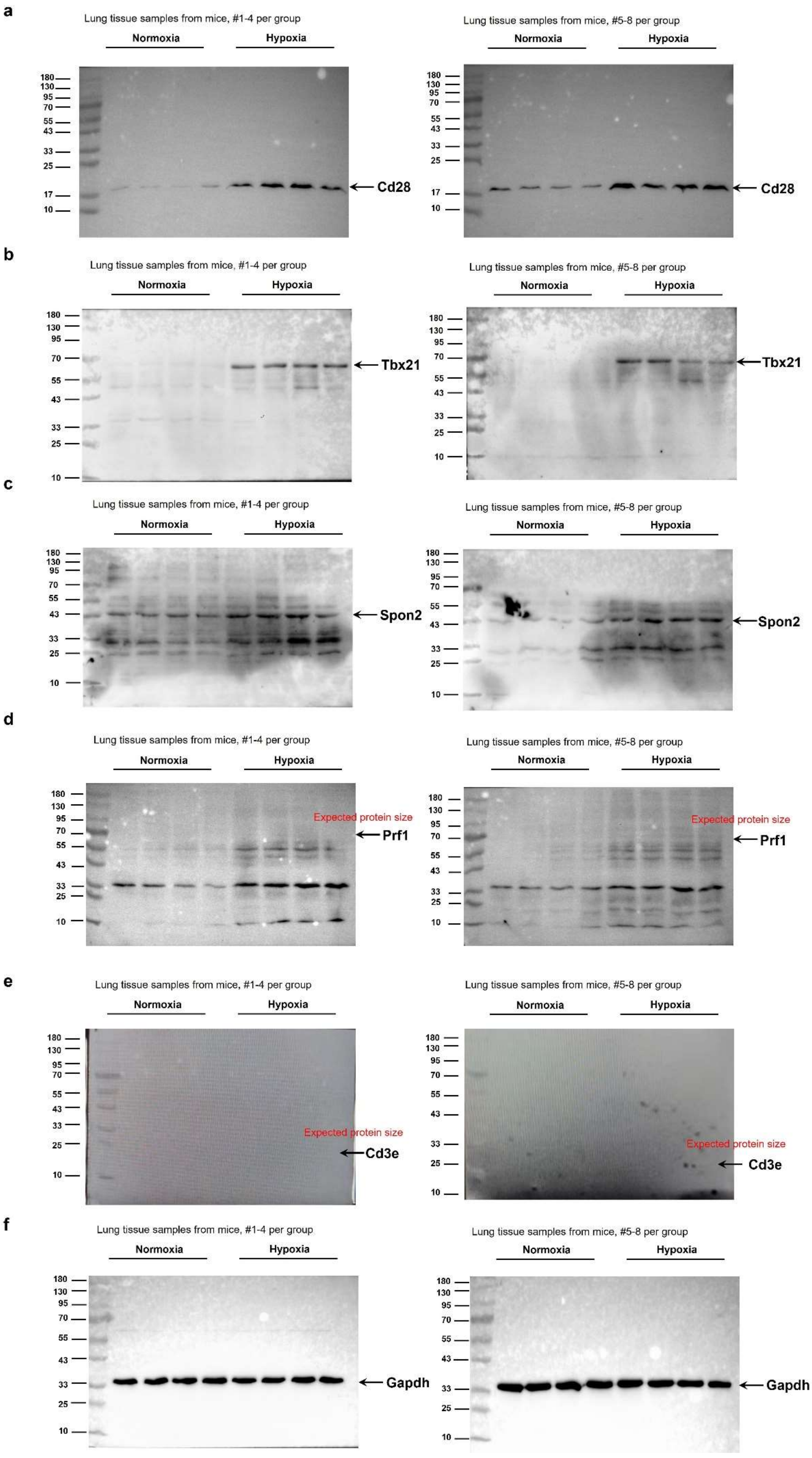
a
Lung tissue samples from mice, #1-4 per group
Normoxia
Hypoxia
180
130
95
70
55
43
33
25
17
10
Cd28
Lung tissue samples from mice, #5-8 per group
Normoxia
Hypoxia
Cd28
b
Lung tissue samples from mice, #1-4 per group
Normoxia
Hypoxia
Tbx21
Lung tissue samples from mice, #5-8 per group
Normoxia
Hypoxia
Tbx21
c
Lung tissue samples from mice, #1-4 per group
Normoxia
Hypoxia
Spon2
Lung tissue samples from mice, #5-8 per group
Normoxia
Hypoxia
Spon2
d
Lung tissue samples from mice, #1-4 per group
Normoxia
Hypoxia
Expected protein size
Prf1
Lung tissue samples from mice, #5-8 per group
Normoxia
Hypoxia
Expected protein size
Prf1
e
Lung tissue samples from mice, #1-4 per group
Normoxia
Hypoxia
Expected protein size
Cd3e
Lung tissue samples from mice, #5-8 per group
Normoxia
Hypoxia
Expected protein size
Cd3e
f
Lung tissue samples from mice, #1-4 per group
Normoxia
Hypoxia
Gapdh
Lung tissue samples from mice, #5-8 per group
Normoxia
Hypoxia
Gapdh

Supplementary Figure 12. Validation of protein expression in lung tissues of the mice model. (**a**). Intact uncropped immunoblotting bands for Cd28 expression in lung tissues of control mice and hypoxic PAH mice, corresponding to that in Figure 5f. (**b**). Intact uncropped immunoblotting bands for Tbx21 expression in lung tissues of control mice and hypoxic PAH mice, corresponding to that in Figure 5f. (**c**). Intact uncropped immunoblotting bands for Spon2 expression in lung tissues of control mice and hypoxic PAH mice, corresponding to that in Figure 5f. (**d**). Intact uncropped immunoblotting bands showed that Prf1 protein was not efficiently detected in the mouse lung tissues. (**e**). Intact uncropped immunoblotting bands showed that Cd3e protein was not efficiently detected in the mouse lung tissues. (**f**). Intact uncropped immunoblotting bands for Gapdh expression in lung tissues of control mice and hypoxic PAH mice, corresponding to that in Figure 5f.

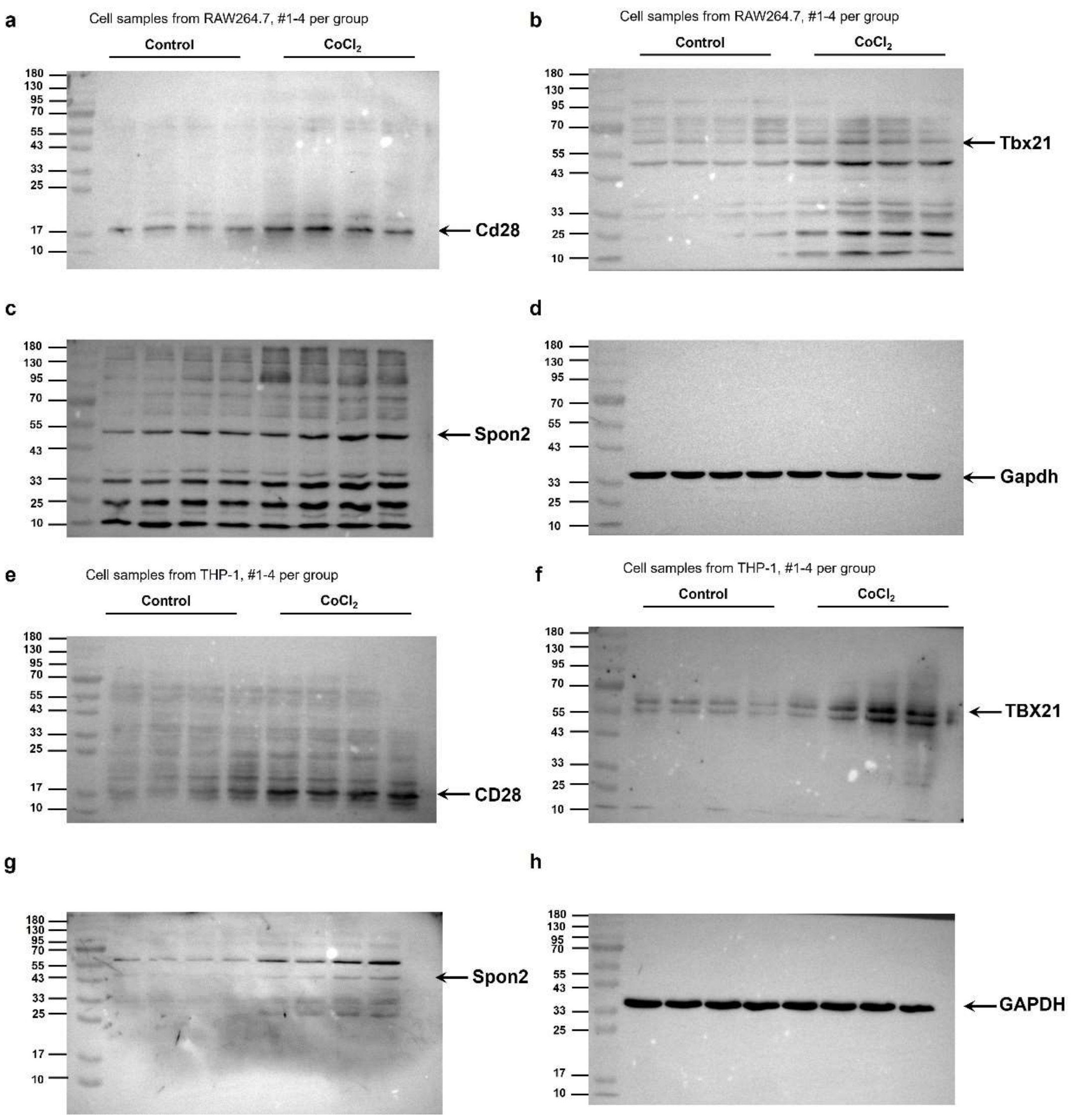

Supplementary Figure 13. Validation of protein expression in RAW264.7 monocytes/macrophages and THP-1 monocytes. (**a-d**) Intact uncropped immunoblotting bands for (**a**) Cd28, (**b**) Tbx21, (**c**) Spon2 and (**d**) Gapdh expression in RAW264.7 monocytes/macrophages in response to $CoCl_2$ (150 μmol/L) or vehicle for 24 h, corresponding to that in Figure 5j. (**e-h**) Intact uncropped immunoblotting bands for (**e**) CD28, (**f**) TBX21, (**g**) SPON2 and (**h**) GAPDH expression in THP-1 monocytes in response to $CoCl_2$ (150 μmol/L) or vehicle for 24 h, corresponding to that in Figure 5k.

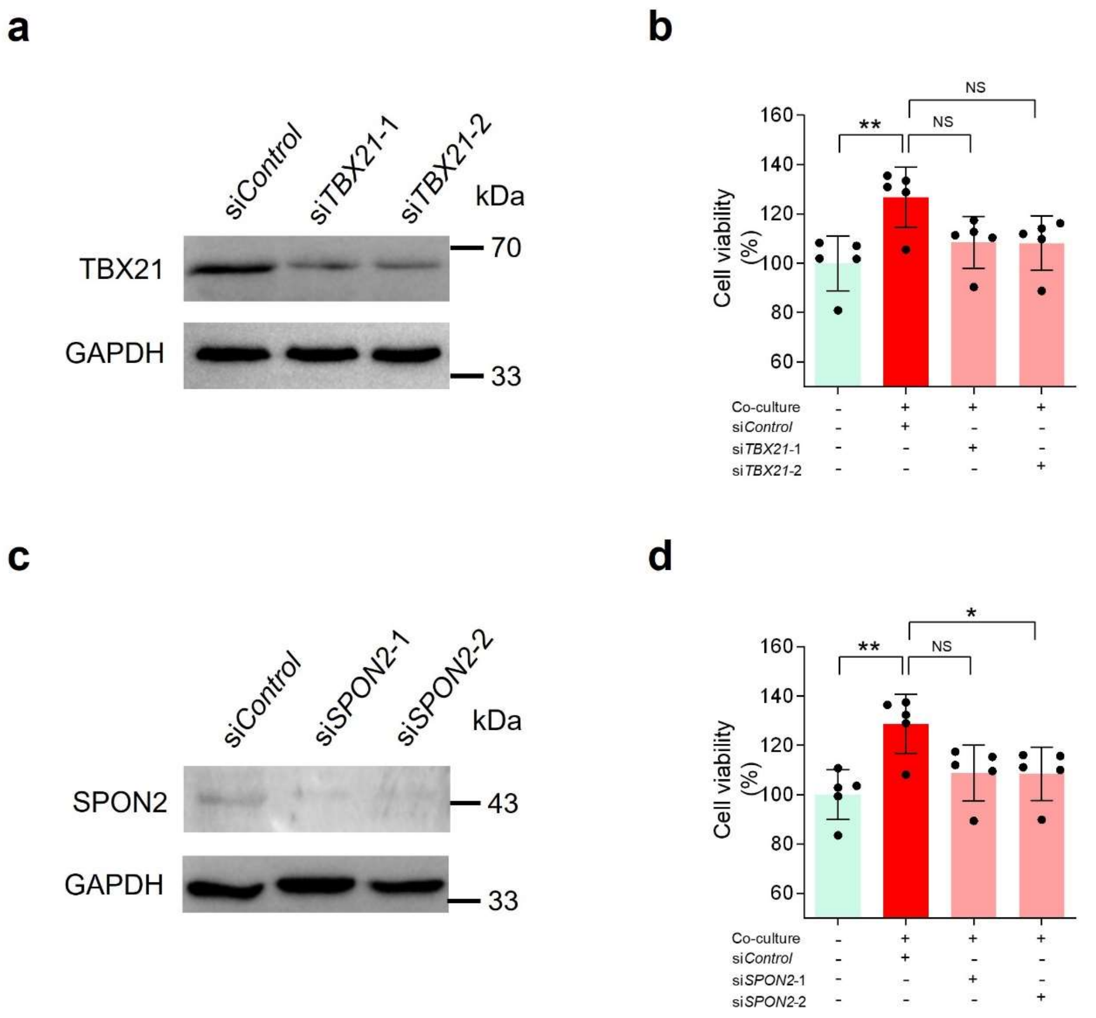

Supplementary Figure 14. Function assessments of *TBX21 and SPON2* using a co-culture model. (**a**) Representative images of immunoblottings of Jurkat Clone E6-1 cells transfected with *TBX21* siRNA (si*TBX21*) or *control* siRNA (si*Control*). (**b**) Viability of human PASMCs co-cultured with or without *TBX21*-silencing or control Jurkat Clone E6-1 cells for 48 h (n = 5 for per group, **p* < 0.05, ***p* < 0.05; one-way ANOVA, Tukey's *post-hoc* test. Data were presented as mean ± SD). (**c**) Representative images of immunoblottings of Jurkat Clone E6-1 cells transfected with *SPON2* siRNA (si*SPON2*) or *control* siRNA (si*Control*). (**d**) Viability of human PASMCs co-cultured with or without *SPON2*-silencing or control Jurkat Clone E6-1 cells for 48 h (n = 5 for per group, **p* < 0.05, ***p* < 0.01; one-way ANOVA, Tukey's *post-hoc* test. Data were presented as mean ± SD).

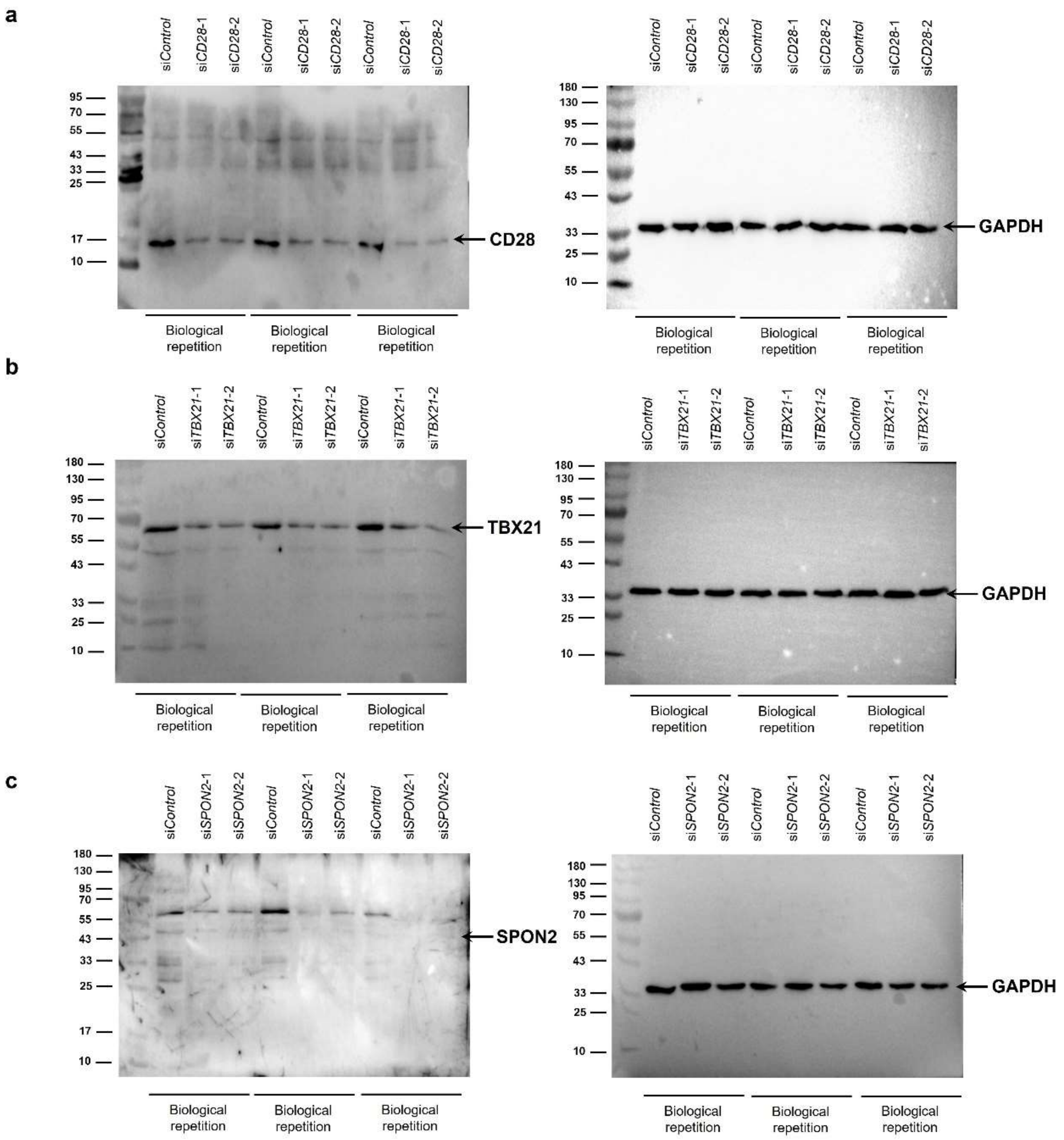

Supplementary Figure 15. **Identification of gene knockdown efficiency.** (**a**) Intact uncropped immunoblotting bands for CD28 (left) and GAPDH (right) expression in Jurkat Clone E6-1 cells, corresponding to that in Figure 5j. (**b**) Intact uncropped immunoblotting bands for TBX21 (left) and GAPDH (right) expression in Jurkat Clone E6-1 cells, corresponding to that in Supplementary Figure 14a. (**c**) Intact uncropped immunoblotting bands for SPON2 (left) and GAPDH (right) expression in Jurkat Clone E6-1 cells, corresponding to that in Supplementary Figure 14c.

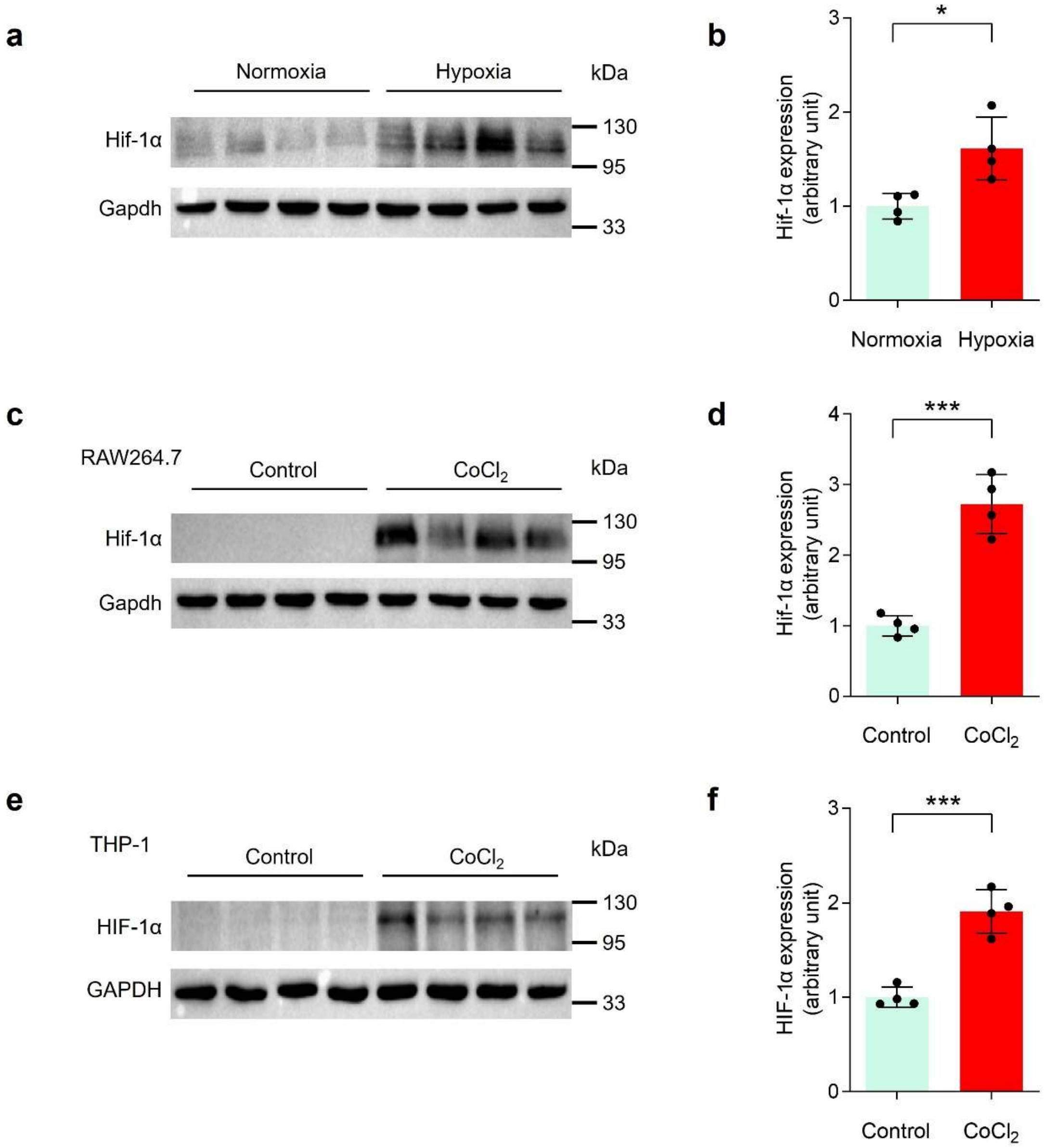

Supplementary Figure 16. Hif-1α (HIF-1α) expression in lung tissues of the PH mice and its expression in response to hypoxia-mimetic agent. (**a, b**) Images of immunoblottings and quantification of the expression of Hif-1α in lung tissues of the control mice and hypoxic PH mice (n = 4 for per group, *p < 0.05; Student's t-test. Data were presented as mean ± SD). (**c, d**) Images of immunoblottings and quantification of the expression of Hif-1α in RAW264.7 monocytes/macrophages responded to $CoCl_2$ (150 μmol/L) or vehicle for 24 h (n = 4 for per group, ***p < 0.001; Student's t-test. Data were presented as mean ± SD). (**e, f**) Images of immunoblottings and quantification of the expression of Hif-1α in THP-1 monocytes responded to $CoCl_2$ (150 μmol/L) or vehicle for 24 h (n = 4 for per group, ***p < 0.001; Student's t-test. Data were presented as mean ± SD).

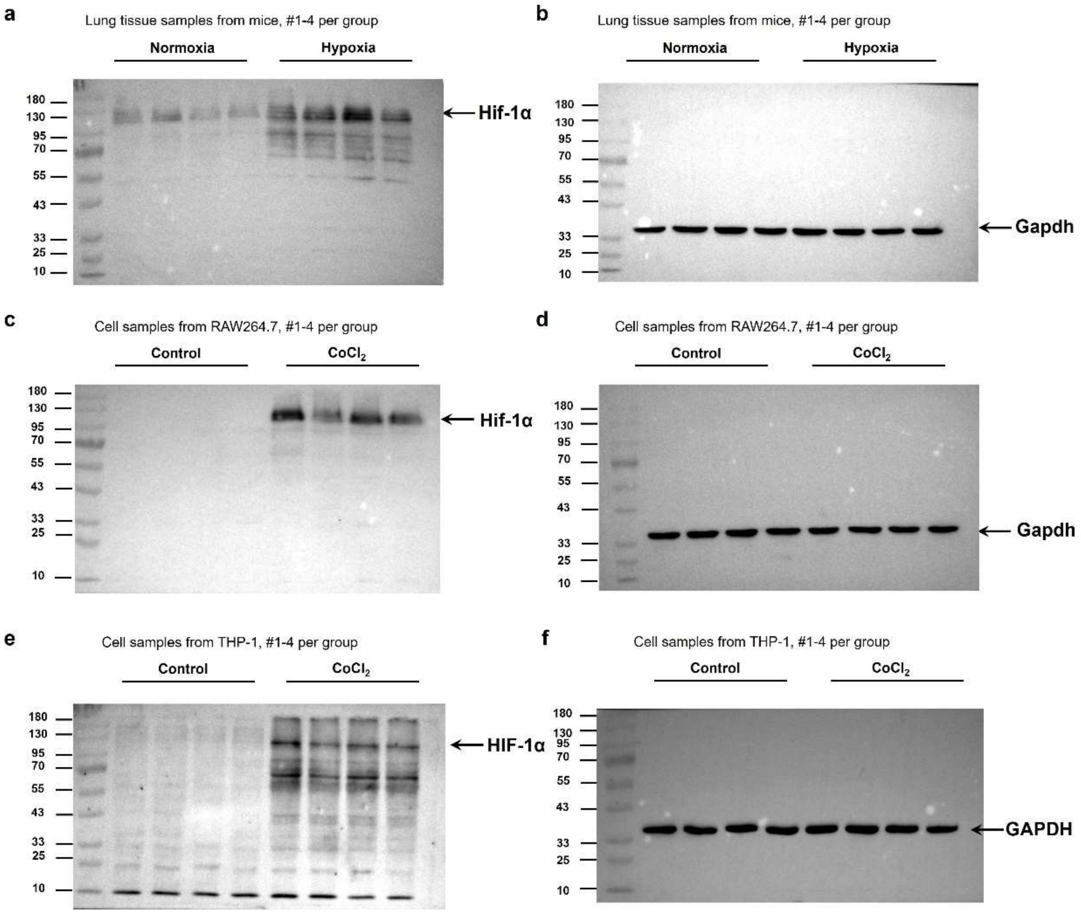

Supplementary Figure 17. Intact uncropped immunoblotting bands for Hif-1α (HIF-1α) expression in lung tissues of the PH mice and cells in response to hypoxia-mimetic agent. (**a, b**) Intact uncropped immunoblotting bands for (**a**) Hif-1α and (**b**) Gapdh expression in lung tissues of the control mice and hypoxic PH mice, corresponding to that in Figure Supplementary Figure 16a. (**c, d**) Intact uncropped immunoblotting bands for (**a**) Hif-1α and (**b**) Gapdh expression in RAW264.7 monocytes/macrophages in response to $CoCl_2$ (150 μmol/L) or vehicle, corresponding to that in Supplementary Figure 16c. (**e**, **f**) Intact uncropped immunoblotting bands for (**a**) HIF-1α and (**b**) GAPDH expression in THP-1 monocytes in response to $CoCl_2$ (150 μmol/L) or vehicle, corresponding to that in Supplementary Figure 16e.

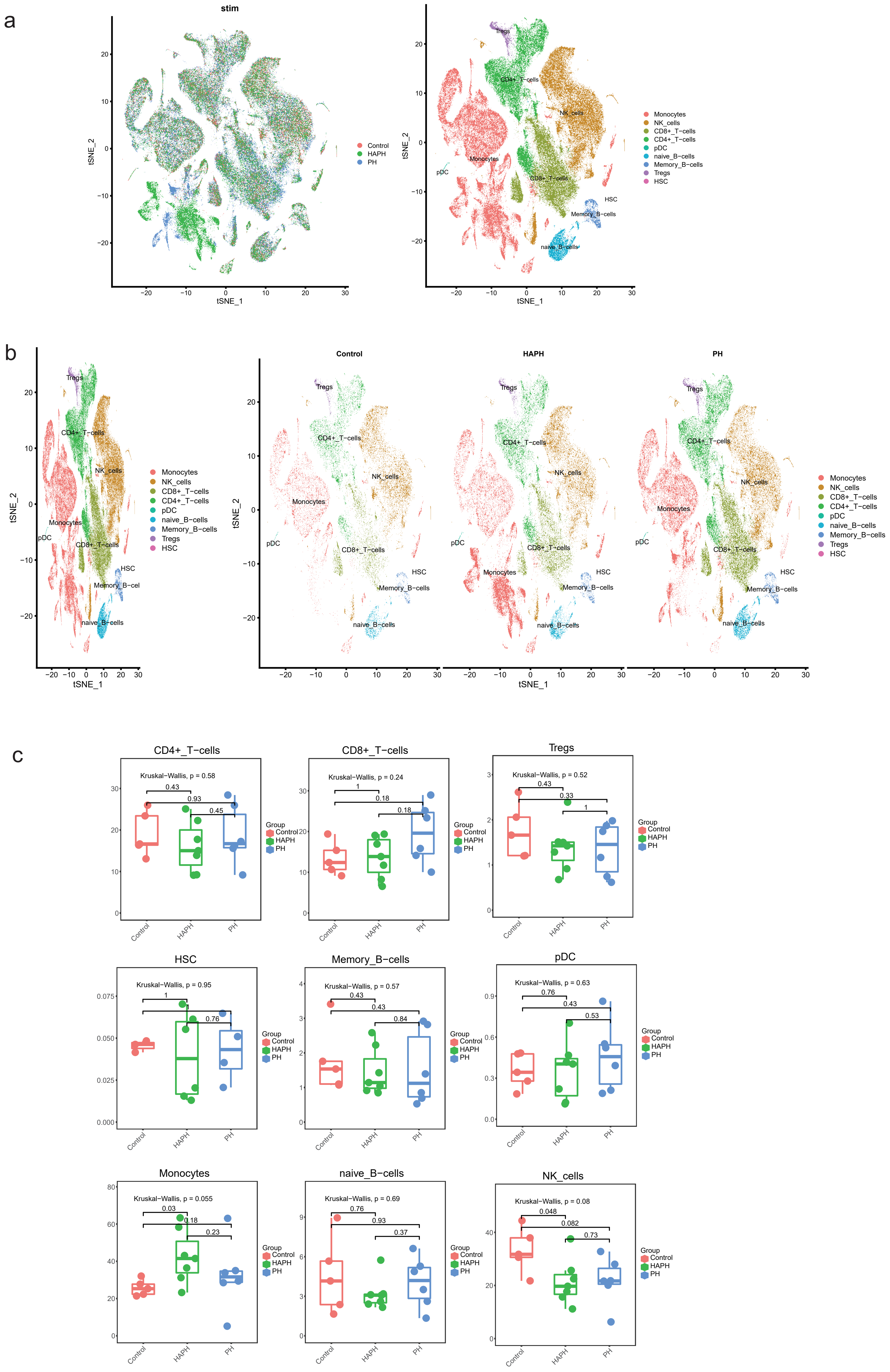

Supplementary Figure 18. Differential immune cell composition in the circulation of patients with HAPH or PH.

Supplementary Figure 18. Differential immune cell composition in the circulation of patients with HAPH or PH. (**a, b**) t-SNE (t-distributed stochastic neighbor embedding) plot of the main immune cell subsets, color-coded for three groups (a, left) or nine clusters (**a**, right), or separate three groups (**b**). (**c**) Boxplots comparing the percentages of indicated cell types in PBMCs between HAPH patients (n=7), PH patients (n=6) and control subjects (n=5). The two-sided *p* values from the Wilcoxon rank-sum test were shown.

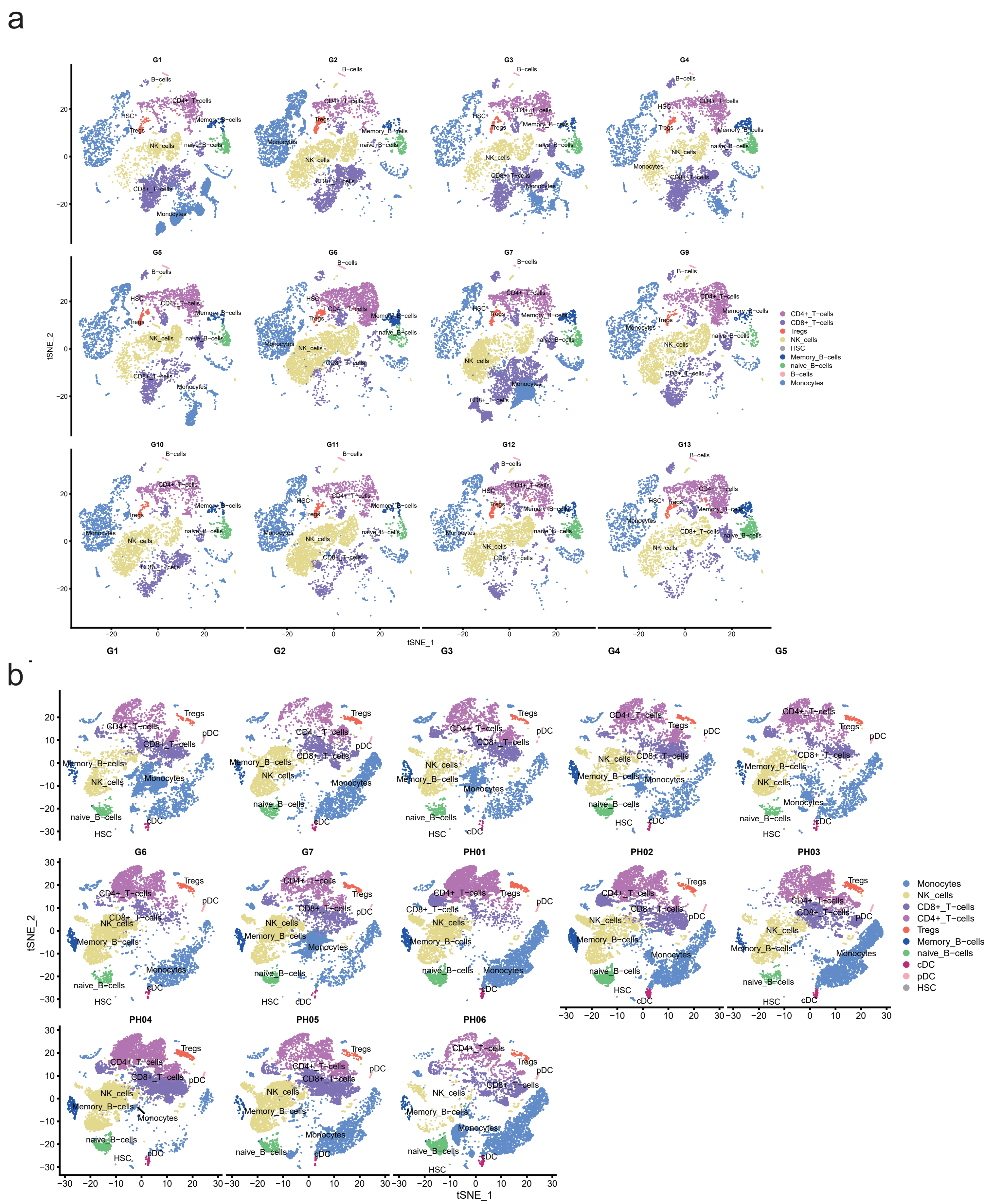

Supplementary Figure 19. tSNE plots of individual samples in HAPH (H) and Control (C) group (a) and in HAPH (H) and PH (P) group (b).

Supplementary Figure 19. tSNE plots of individual samples in HAPH (H) and Control (C) group (**a**) and in HAPH (H) and PH (P) group (**b**).

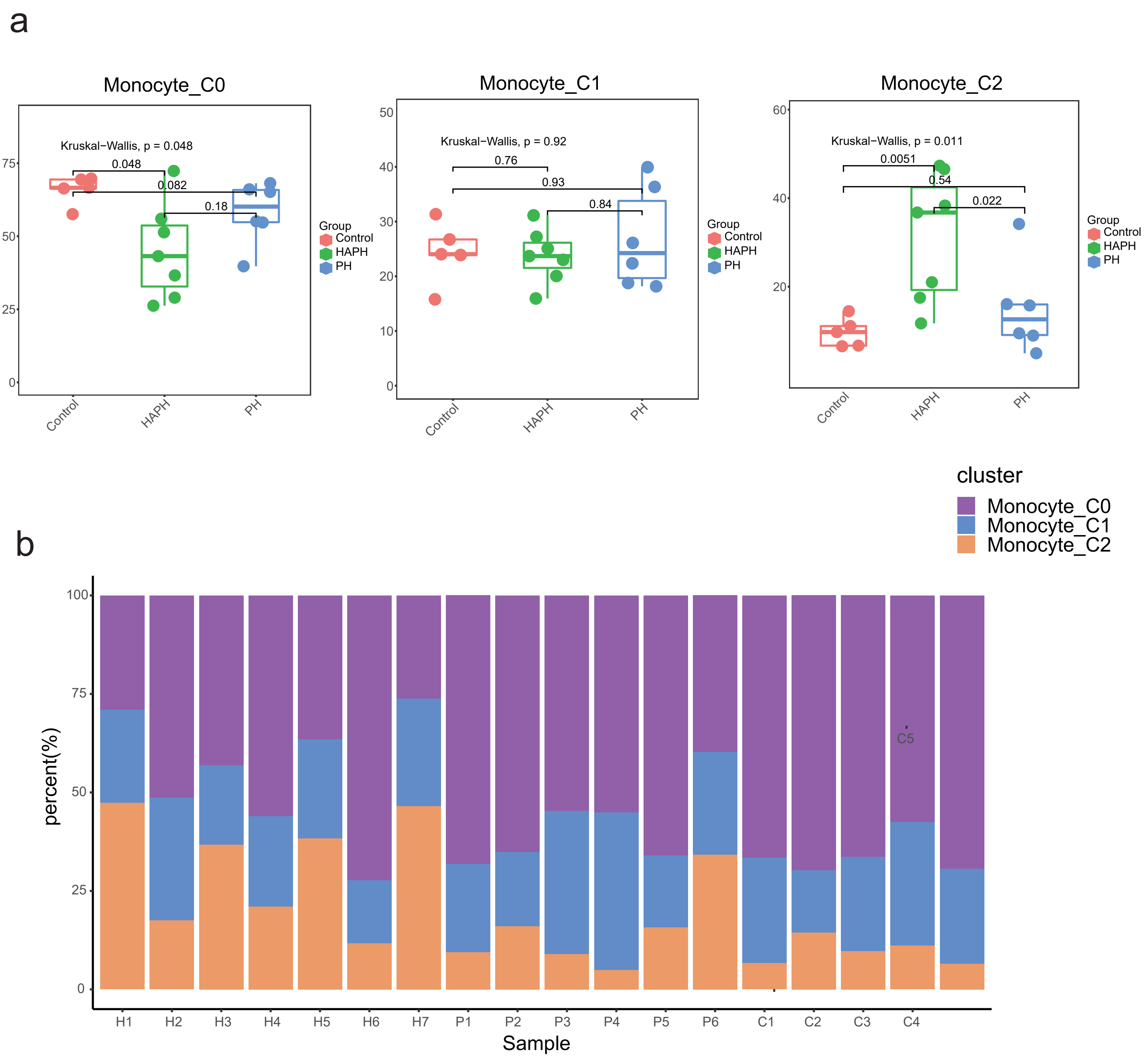

Supplementary Figure 20. Monocyte clusters in PBMCs from patients with HAPH (H), PH (P) and controls (C).

Supplementary Figure 20. Monocyte clusters in PBMCs from patients with HAPH, PH and controls. (**a**) Boxplots comparing the percentages of each monocyte cluster in PBMCs between HAPH patients (n=7), PH patients (n=6) and control subjects (n=5). The two-sided *p* values from the Wilcoxon rank-sum test were shown. (**b**) Proportions of each cell type in HAPH patients (n=7), PH patients (n=6) and control subjects (n=5).
