# Supplementary material for: Single-cell analysis of peripheral blood from high-altitude pulmonary hypertension patients identifies a distinct monocyte phenotype
Source: Nat Commun. 2023 Mar 31;14:1820. doi: 10.1038/s41467-023-37527-4 (PMC10066231; doi:10.1038/s41467-023-37527-4)
Supplement: Supplementary file 3 — Description of Additional Supplementary Files [file 41467_2023_37527_MOESM3_ESM.pdf]

Description of Additional Supplementary Files

Supplementary Data 1: The differential expression gene list of monocyte subsets (C0) in comparison of cases (HAPH) and controls.

Supplementary Data 2: The differential expression gene list of monocyte subsets (C1) in comparison of cases (HAPH) and controls.

Supplementary Data 3: The differential expression gene list of monocyte subsets (C2) in comparison of cases (HAPH) and controls.
