# Supplementary material for: Single-cell analysis of peripheral blood from high-altitude pulmonary hypertension patients identifies a distinct monocyte phenotype
Source: Nat Commun. 2023 Mar 31;14:1820. doi: 10.1038/s41467-023-37527-4 (PMC10066231; doi:10.1038/s41467-023-37527-4)
Supplement: Supplementary file 7 — Reporting Summary [file 41467_2023_37527_MOESM7_ESM.pdf]

nature portfolio

Corresponding author(s): Zhi-Cheng Jing, Hong Liu

Last updated by author(s): Feb 20, 2023

Reporting Summary

Nature Portfolio wishes to improve the reproducibility of the work that we publish. This form provides structure for consistency and transparency in reporting. For further information on Nature Portfolio policies, see our Editorial Policies and the Editorial Policy Checklist.

Statistics

For all statistical analyses, confirm that the following items are present in the figure legend, table legend, main text, or Methods section.

n/a	Confirmed	
☐	☒	The exact sample size (*n*) for each experimental group/condition, given as a discrete number and unit of measurement
☐	☒	A statement on whether measurements were taken from distinct samples or whether the same sample was measured repeatedly
☐	☒	The statistical test(s) used AND whether they are one- or two-sided<br>*Only common tests should be described solely by name; describe more complex techniques in the Methods section.*
☐	☒	A description of all covariates tested
☐	☒	A description of any assumptions or corrections, such as tests of normality and adjustment for multiple comparisons
☐	☒	A full description of the statistical parameters including central tendency (e.g. means) or other basic estimates (e.g. regression coefficient) AND variation (e.g. standard deviation) or associated estimates of uncertainty (e.g. confidence intervals)
☐	☒	For null hypothesis testing, the test statistic (e.g. *F*, *t*, *r*) with confidence intervals, effect sizes, degrees of freedom and *P* value noted<br>*Give P values as exact values whenever suitable.*
☒	☐	For Bayesian analysis, information on the choice of priors and Markov chain Monte Carlo settings
☒	☐	For hierarchical and complex designs, identification of the appropriate level for tests and full reporting of outcomes
☐	☒	Estimates of effect sizes (e.g. Cohen's *d*, Pearson's *r*), indicating how they were calculated

Our web collection on statistics for biologists contains articles on many of the points above.

Software and code

Policy information about availability of computer code

Data collection	10X Genomics Chromium single-cell controller and single cell 5' library and gel beads kit and single cell A chip kit were used to acquire raw sequencing data.
Data analysis	CellRanger (v 4.0.0) was used to process raw sequencing data in single cell level. Seurat R package (v3.2) was used to perform the quality control, clustering and differential expression analysis. clusterProfiler (v3.10.1) was used to perform functional interaction network. GSEA application (v2.2.2.4) and MSigDB (v6.2) was used to functional enrichment. CellPhoneDB (v2.0) was used to perform cell cell imunications. Illustration was used custom code conducted by R language. Data analysis of in vivo and vitro studies were applied Quantity One 1-D software (v4.6.6, PC), GraphPad Prism (v5.0). Flow cytometer data were analyzed using FlowJo (v10.4).

For manuscripts utilizing custom algorithms or software that are central to the research but not yet described in published literature, software must be made available to editors and reviewers. We strongly encourage code deposition in a community repository (e.g. GitHub). See the Nature Portfolio guidelines for submitting code & software for further information.

Data

Policy information about availability of data

All manuscripts must include a data availability statement. This statement should provide the following information, where applicable:

- Accession codes, unique identifiers, or web links for publicly available datasets
- A description of any restrictions on data availability
- For clinical datasets or third party data, please ensure that the statement adheres to our policy

The data supporting the findings from this study are available in the main manuscript and supplementary materials. The raw sequence data reported in this paper have been deposited in the GenomeSequence Archive of the Beijing Institute of Genomics (BIG) Data Center, BIG,Chinese Academy of Sciences (GSA-Human: RA002501) and are publicly accessible at https://ngdc.cncb.ac.cn/gsa-human/browse/HRA002501. Any other raw data or non-commercial material used in this study are available from the corresponding author upon request. Source data are provided with this paper.

Human research participants

Policy information about studies involving human research participants and Sex and Gender in Research.

Reporting on sex and gender	The sex and gender were not considered in the study design.
Population characteristics	Multicentered data was collected. In First Affiliated Hospital of Dali University, a total of 248 Naxi villagers were selected from Gaomei Village (3194.97 m), Mushu Village (3109.72 m) and Lutu Village (2981.71 m) in Qihe Town, Lijiang City, Yunnan Province. 11 subjects with systolic PAP (sPAP) ≥40 mmHg were finally considered as HAPH patients and recruited in our study. At the same time, 15 contemporary individuals who lived at the same altitude and were not relatives within three generations were recruited as controls. In Peking Union Medical College Hospital, 6 PH cases living at 45 m altitude were also enrolled. Candidates with left-heart disease, lung disease, and renal insufficiency were excluded.
Recruitment	see above.
Ethics oversight	This study was approved by the Ethics Committees of the First Affiliated Hospital of Dali University and Peking Union Medical College Hospital, and the relevant informed consent documents were signed by the subjects before sample collection and data acquisition.

Note that full information on the approval of the study protocol must also be provided in the manuscript.

Field-specific reporting

Please select the one below that is the best fit for your research. If you are not sure, read the appropriate sections before making your selection.

☒ Life sciences ☐ Behavioural & social sciences ☐ Ecological, evolutionary & environmental sciences

For a reference copy of the document with all sections, see nature.com/documents/nr-reporting-summary-flat.pdf

Life sciences study design

All studies must disclose on these points even when the disclosure is negative.

Sample size	For experiments involving gene-function validation in hypoxic PH mice, n=7 was chosen as the minimal replicate number (independent sample). For experiments involving gene-function validation in PASMCs, n=5 was chosen as the minimal replicate number. And sample size was determined with reference to our previous similar study design (Sci Adv. 2020 Dec 9;6(50):eaba2470; Eur Respir J. 2020 Nov 5;56 (5):2000522).
Data exclusions	Data were not excluded from analysis.
Replication	All experiments except for single cell sequencing and flow cytometry were done in replicate, and all attempts at replication were successful.
Randomization	Animals were randomly separated to experimental and control groups. Random fields from each slide and random sections from mice were used to calculate the wall area/total vessel area for H&E staining. For other in vitro experiments, samples were randomly grouped into experimental groups and controls.
Blinding	Animals phenotypes such as right heart catheterization and right heart hypertrophy index were determined in a blinded fashion. Blind method was also used for data collection and analysis involving flow cytometry, immunoblotting, real time PCR and cell viability determination.

Behavioural & social sciences study design

All studies must disclose on these points even when the disclosure is negative.

Study description	*Briefly describe the study type including whether data are quantitative, qualitative, or mixed-methods (e.g. qualitative cross-sectional, quantitative experimental, mixed-methods case study).*
Research sample	*State the research sample (e.g. Harvard university undergraduates, villagers in rural India) and provide relevant demographic information (e.g. age, sex) and indicate whether the sample is representative. Provide a rationale for the study sample chosen. For studies involving existing datasets, please describe the dataset and source.*
Sampling strategy	*Describe the sampling procedure (e.g. random, snowball, stratified, convenience). Describe the statistical methods that were used to predetermine sample size OR if no sample-size calculation was performed, describe how sample sizes were chosen and provide a rationale for why these sample sizes are sufficient. For qualitative data, please indicate whether data saturation was considered, and what criteria were used to decide that no further sampling was needed.*
Data collection	*Provide details about the data collection procedure, including the instruments or devices used to record the data (e.g. pen and paper, computer, eye tracker, video or audio equipment) whether anyone was present besides the participant(s) and the researcher, and whether the researcher was blind to experimental condition and/or the study hypothesis during data collection.*
Timing	*Indicate the start and stop dates of data collection. If there is a gap between collection periods, state the dates for each sample cohort.*
Data exclusions	*If no data were excluded from the analyses, state so OR if data were excluded, provide the exact number of exclusions and the rationale behind them, indicating whether exclusion criteria were pre-established.*
Non-participation	*State how many participants dropped out/declined participation and the reason(s) given OR provide response rate OR state that no participants dropped out/declined participation.*
Randomization	*If participants were not allocated into experimental groups, state so OR describe how participants were allocated to groups, and if allocation was not random, describe how covariates were controlled.*

Ecological, evolutionary & environmental sciences study design

All studies must disclose on these points even when the disclosure is negative.

Study description	*Briefly describe the study. For quantitative data include treatment factors and interactions, design structure (e.g. factorial, nested, hierarchical), nature and number of experimental units and replicates.*
Research sample	*Describe the research sample (e.g. a group of tagged Passer domesticus, all Stenocereus thurberi within Organ Pipe Cactus National Monument), and provide a rationale for the sample choice. When relevant, describe the organism taxa, source, sex, age range and any manipulations. State what population the sample is meant to represent when applicable. For studies involving existing datasets, describe the data and its source.*
Sampling strategy	*Note the sampling procedure. Describe the statistical methods that were used to predetermine sample size OR if no sample-size calculation was performed, describe how sample sizes were chosen and provide a rationale for why these sample sizes are sufficient.*
Data collection	*Describe the data collection procedure, including who recorded the data and how.*
Timing and spatial scale	*Indicate the start and stop dates of data collection, noting the frequency and periodicity of sampling and providing a rationale for these choices. If there is a gap between collection periods, state the dates for each sample cohort. Specify the spatial scale from which the data are taken*
Data exclusions	*If no data were excluded from the analyses, state so OR if data were excluded, describe the exclusions and the rationale behind them, indicating whether exclusion criteria were pre-established.*
Reproducibility	*Describe the measures taken to verify the reproducibility of experimental findings. For each experiment, note whether any attempts to repeat the experiment failed OR state that all attempts to repeat the experiment were successful.*
Randomization	*Describe how samples/organisms/participants were allocated into groups. If allocation was not random, describe how covariates were controlled. If this is not relevant to your study, explain why.*
Blinding	*Describe the extent of blinding used during data acquisition and analysis. If blinding was not possible, describe why OR explain why blinding was not relevant to your study.*

Did the study involve field work? ☐ Yes ☐ No

Field work, collection and transport

Field conditions	*Describe the study conditions for field work, providing relevant parameters (e.g. temperature, rainfall).*
Location	*State the location of the sampling or experiment, providing relevant parameters (e.g. latitude and longitude, elevation, water depth).*
Access & import/export	*Describe the efforts you have made to access habitats and to collect and import/export your samples in a responsible manner and in compliance with local, national and international laws, noting any permits that were obtained (give the name of the issuing authority, the date of issue, and any identifying information).*
Disturbance	*Describe any disturbance caused by the study and how it was minimized.*

Reporting for specific materials, systems and methods

We require information from authors about some types of materials, experimental systems and methods used in many studies. Here, indicate whether each material, system or method listed is relevant to your study. If you are not sure if a list item applies to your research, read the appropriate section before selecting a response.

Materials & experimental systems

n/a	Involved in the study
☐	☒ Antibodies
☐	☒ Eukaryotic cell lines
☒	☐ Palaeontology and archaeology
☐	☒ Animals and other organisms
☐	☒ Clinical data
☒	☐ Dual use research of concern

Methods

n/a	Involved in the study
☒	☐ ChIP-seq
☐	☒ Flow cytometry
☒	☐ MRI-based neuroimaging

Antibodies

Antibodies used	Anti-Spon2 antibody [EPR9799] (abcam, #ab171955, 1:1000 dilution), anti-Prf1 antibody [E7D8R] (Cell Signaling Technology, #62550, 1:1000 dilution), anti-Tbx21 antibody [E4I2K] (Cell Signaling Technology, #97135, 1:1000 dilution), anti-Cd28 antibody [D2Z4E] (Cell Signaling Technology, #38774, 1:1000 dilution), anti-Cd3e antibody [CD3-12] (Cell Signaling Technology, #4443, 1:1000 dilution), anti-Hif-1α [EPR16897] (abcam, #ab179483, 1:1000 dilution), anti-glyceraldehyde-3-phosphate dehydrogenase (Gapdh) monoclonal antibody [D16H11] (Cell Signaling Technology, #5174, 1:1000 dilution). CD3-FITC [HIT3a] (BD Biosciences, #555339, 1:20 dilution), CD4-APC-CY7 [SK3] (BD Biosciences, #341115, 1:40 dilution), CD8-PerCP [SK3] (Biolegend, #344708, 1:40 dilution), CD16-PE-CF594 [3G8] (BD Biosciences, #562293, 1:40 dilution), CD56-PE-CY7 [Y1/82A] (Biolegend, #333816, 1:40 dilution), CD14-PE [M5E2] (Biolegend, #301806, 1:40 dilution), HIF1α-FITC [546-16] (Biolegend, #359708, 1:40 dilution), VEGFA antibody [EP1176Y] (Abcam, #ab52917, 1:30 dilution), Goat Anti-Rabbit IgG H&L-AF405/DAPI (Abcam, #ab175652, 1:2000 dilution).
Validation	All antibodies in this study were commercially purchased and have been validated by the vendors for species and application. Validation data are available from the respective vendor's respective websites.

Eukaryotic cell lines

Policy information about cell lines and Sex and Gender in Research

Cell line source(s)	Jurkat Clone E6-1 T cells were purchased from Procell Life Science & Technology Co., Ltd. (Wuhan, China) and Human pulmonary artery smooth muscle cells (PASMCs) were purchased from ScienCell Research Laboratories.
Authentication	Jurkat Clone E6-1 T was identified by Procell Life Science & Technology Co., Ltd. (Wuhan, China), and STR typing of cell line DNA showed that it completely matched the cell type in the cell bank, and no cross-contamination was found.
Mycoplasma contamination	Jurkat Clone E6-1 T was identified by Procell Life Science & Technology Co., Ltd. (Wuhan, China), and mycoplasma contamination was not found.
Commonly misidentified lines (See ICLAC register)	No commonly misidentified cell lines were used.

Animals and other research organisms

Policy information about studies involving animals; ARRIVE guidelines recommended for reporting animal research, and Sex and Gender in Research

Laboratory animals	Adult C57BL/6 mice (8 weeks old, 18–22 g, half males and half females) were purchased from Charles River Laboratories (Beijing, China).
Wild animals	No wild animals were used in this study.
Reporting on sex	Gender was not considered in this study design. We adopted the method of half males and half females (20 mice in total), and did not find that gender had a significant effect on the right ventricular systolic pressure, the degree of pulmonary artery remodeling, or the expression level of the target protein in the mice.
Field-collected samples	No field-collected samples were used in this study.
Ethics oversight	All procedures involving mice were approved by the Animal Care and Use Committee of Peking Union Medical College Hospital, Chinese Academy of Medical Sciences. The experiment was carried out in accordance with the Guideline for Care and Use of Laboratory Animals published by the US National Institutes of Health, and the Guidelines for the ethical review of laboratory animal welfare People's Republic of China National Standard GB/T 35892-2018.

Note that full information on the approval of the study protocol must also be provided in the manuscript.

Clinical data

Policy information about clinical studies

All manuscripts should comply with the ICMJEguidelines for publication of clinical research and a completedCONSORT checklist must be included with all submissions.

Clinical trial registration	N/A
Study protocol	ScRNA-seq analyses were performed to compare the differences in PBMCs between 7 cases of high-altitude pulmonary hypertension (HAPH) and 5 controls, and also between 7 HAPH and 6 cases of pulmonary hypertension(PH). Validation studies in PBMCs were carried out among 6 PH,11 HAPH and 10 controls with fluorescence-activated cell sorting (FACS) analysis.
Data collection	11 patients with systolic PAP (sPAP) ≥40 mmHg were recruited as HAPH patients from Gaomei Village (3194.97 m), Mushu Village (3109.72 m) and Lutu Village (2981.71 m) in Qihe Town, Lijiang City, Yunnan Province. 15 contemporary individuals who lived at the same altitude and were not relatives within three generations were recruited as controls. In Peking Union Medical College Hospital, 6 PH cases living at 45 m altitude were also enrolled. All the participants were recuited from July 2021 to February 2022.
Outcomes	N/A

Flow Cytometry

Plots

Confirm that:

- [x] The axis labels state the marker and fluorochrome used (e.g. CD4-FITC).
- [x] The axis scales are clearly visible. Include numbers along axes only for bottom left plot of group (a 'group' is an analysis of identical markers).
- [x] All plots are contour plots with outliers or pseudocolor plots.
- [x] A numerical value for number of cells or percentage (with statistics) is provided.

Methodology

Sample preparation	Peripheral blood were extracted from patients of HAPH (10 samples), PH (6 samples) and Healthy donor （Control, 10 samples） and then subjected to density gradient centrifugation (Ficoll-Hypaque, TBD Science, Tianjin, China) for 20 min at 24°C and 2000 rpm. The cells at the Ficoll interface were collected, diluted in PBS, and centrifuged for 8 min at 1500 rpm.
Instrument	BD AriaII Cell Sorting System (3 Lasers)
Software	FlowJo(V10.4)
Cell population abundance	The proportions of T lymphocytes (CD4+ T and CD8+ T), NK cells and Monocytes and their subpopulations (C0, C1 and C2) detected by PBMC are shown in Figure 3, 7 and Supplemental files.
Gating strategy	Total lymphocytes or NKs or Monocytes were initially gated based on expression of the specifical antigen CD3 (Lymphocyte), CD16 (NKs) and CD68 (Monocytes) respectively. CD3-FITC for total lymphocytes labeling, CD4-APC-CY7 and CD8-PerCP for CD4+ or CD8+ T lymphocytes labeling. CD3-FITC negativity gates for NK cell populations, CD16-PE-CF594 and CD56-PE-CY7 for NKs labeling. CD68-PE-CY7 for total Monocytes labeling. CD16-PE-CF594 and CD14-PE for monocyte subpopulations labeling:

C0, C1 and C2.

☒ Tick this box to confirm that a figure exemplifying the gating strategy is provided in the Supplementary Information.
